# Endothelium-specific endoglin triggers astrocyte reactivity via extracellular vesicles in a mouse model of Alzheimer’s disease

**DOI:** 10.1186/s13024-025-00875-4

**Published:** 2025-07-23

**Authors:** Pingao Zhang, Chenghuan Song, Jiyun Shi, Zijie Wei, Jing Wang, Wanying Huang, Rui Zhang, Jintao Wang, Xiaoli Yang, Gang Wang, Xiaoling Gao, Yongfang Zhang, Hongzhuan Chen, Hao Wang

**Affiliations:** 1 Department of Pharmacology and Chemical Biology, Shanghai Jiao Tong University School of Medicine, Shanghai, 200025 China; 2 Academy of Integrative Medicine, Shanghai University of Traditional Chinese Medicine, Shanghai, 201203 China; 3 Department of Neurology, Ruijin Hospital, Shanghai Jiao Tong University School of Medicine, Shanghai, 200025 China; 4 Department of Neurology, Affiliated Hospital of Hebei University of Engineering, Handan, 056002 China; 5 Department of Neurology, Renji Hospital, Shanghai Jiao Tong University School of Medicine, Shanghai, 200025 China; 6 Lab for Future Health, Frontier Center of TCM Chemobiology, Shuguang Hospital, Shanghai University of Traditional Chinese Medicine, Shanghai, 201203 China

**Keywords:** Alzheimer's disease, Neurovascular unit, Brain microvascular endothelial cells, Astrocyte, Cerebrovascular endothelial extracellular vesicles, Endoglin

## Abstract

**Background:**

Alzheimer’s disease (AD) is a multifaceted neurodegenerative disorder with a complex etiology that extends beyond the well-documented amyloid-β and tau pathologies. Growing evidence implicates cerebrovascular dysfunction, particularly brain microvascular endothelial cells (BMECs) dysfunction, as an early contributor to AD pathogenesis. However, how BMECs influence on neighboring astrocytes needs to be further explored.

**Methods:**

We employed a multi-omics approach integrating bulk RNA sequencing of human BMECs with proteomic analysis of cerebrospinal fluid (CSF) from AD patients and cerebrovascular endothelial extracellular vesicles (CEEVs). The role of identified candidate proteins was investigated in vitro and in vivo utilizing CEEVs transplantation and BMEC-astrocyte co-cultures. Endothelial cell-specific knockdown or treatment with a monoclonal antibody was used to assess the functional consequences on cognitive impairment and AD pathology via two-photon imaging and behavioral experiments on APP/PS1 mice.

**Results:**

The elevated endothelium-specific protein Endoglin (ENG) was identified in the brain and serum of AD individuals and APP/PS1 mice, and the supernatant of injured BMECs. ENG was released and delivered to adjacent astrocytes via CEEVs, and subsequently upregulated TGFBRI/Smad3 pathway in astrocytes, leading to astrocyte reactivity and the release of pro-inflammatory cytokines. Endothelial cell-specific ENG knockdown or treating with ENG monoclonal antibody Carotuximab significantly suppressed reactive astrocytes, reduced neuroinflammation, and improved cognitive performance of APP/PS1 mice.

**Conclusions:**

This study reveals a novel mechanism by which BMECs-derived ENG, delivered via CEEVs, drives astrocyte reactivity. These findings redefine the role of cerebrovascular dysfunction in AD pathogenesis and identify ENG as both a potential biomarker and a promising therapeutic target for AD.

**Supplementary Information:**

The online version contains supplementary material available at 10.1186/s13024-025-00875-4.

## Background

Alzheimer’s disease (AD), the leading cause of dementia worldwide, presents a formidable challenge to global health. Although the amyloid cascade hypothesis has dominated AD research for decades, the incomplete efficacy of amyloid-targeting therapies underscores the need for a more comprehensive understanding of AD pathogenesis. Emerging evidence strongly implicates cerebrovascular dysfunction as a critical early driver of AD progression, preceding and potentially initiating the characteristic amyloid and tau pathologies [1, 2]. This paradigm shift is supported by compelling preclinical and clinical data demonstrating that vascular pathology, including blood-brain barrier (BBB) disruption, cerebral microbleeds, and elevated cerebrovascular resistance, not only correlates with but actively contributes to neuronal loss and cognitive decline [1, 2]. Furthermore, the observed association between cerebrovascular dysfunction and reduced cerebral glucose metabolism, increased amyloid burden and elevated neurodegenerative biomarkers (p-tau, neurofilament light chain, GFAP) [3], strongly suggests a central role for cerebrovascular impairment, either independently or synergistically with amyloid and tau pathologies, in the initiation and progression of AD. Despite this growing body of evidence, the precise molecular mechanisms orchestrating the interplay between cerebrovascular injury and the neurodegenerative cascade remain elusive.

The traditional view regarding brain microvascular endothelial cells (BMECs) as passive participants in the neurovascular unit (NVU) is increasingly being challenged. Recent studies provide compelling evidence that BMECs actively regulate NVU homeostasis, particularly neighboring astrocytes, under vascular stress [4–6]. For instance, vascular injuries caused by hypertension or stroke elicit astrocyte reactivity [4, 5]. This phenomenon, along with parallels between vascular damage and astrocyte activation in AD [7], strongly suggests direct communication between BMECs and astrocytes.

Astrocytes, the most abundant glial cells in the brain, are strategically located at the interface between neurons and blood vessels, positioning them as critical mediators of neurovascular coupling and respondents to cerebrovascular insults. In the context of AD, astrocyte reactivity, characterized by a complex shift in their gene expression profile, cytokine release, and interactions with neurons and blood vessels, contributes significantly to neuroinflammation, synaptic dysfunction, and neuronal loss [8, 9]. While amyloid-beta (Aβ) has been implicated as a trigger for astrocyte reactivity [10, 11], the presence of robust astrocyte reactivity in preclinical stages of AD, even in the individuals with mild cognitive impairment (MCI) [12, 13], suggests that additional upstream factors initiate this detrimental glial response, potentially emanating from the cerebrovascular damage.

This study uncovers a novel mechanism by which injured BMECs initiate a cascade of events leading to astrocyte reactivity and neurodegeneration in AD. Utilizing an innovative multi-omics approach integrating bulk RNA sequencing data from human BMECs with proteomic analyses of cerebrospinal fluid (CSF) from AD patients and cerebrovascular endothelial extracellular vesicles (CEEVs), we identified Endoglin (ENG), a transmembrane protein, as a critical endothelial cell-specific regulator of astrocyte reactivity. The expression of ENG was elevated in the brain and serum of both AD patients and APP/PS1 transgenic mice, correlating with disease severity. Mechanistically, ENG, released via extracellular vesicles (EVs) from injured BMECs, acted as a potent modulator of TGFβ signaling in astrocytes, promoting astrocyte reactivity and subsequent neuroinflammation. Importantly, targeted suppression of ENG, both through endothelial cell-specific knockdown and treatment with ENG monoclonal antibody Carotuximab, effectively suppressed reactive astrocytes, ameliorated neuroinflammation, and significantly improved cognitive function in APP/PS1 mice. Our findings elucidate a previously undescribed mechanism governing BMEC-astrocyte interaction, revealing CEEVs-mediated delivery of ENG as a critical modulator. This novel pathway significantly reframes our understanding of the complex interplay between cerebrovascular compromise and neurodegeneration, offering a promising therapeutic target for AD.

## Results

### Vascular injury contributes to cognitive impairment and astrocyte reactivity

**Fig. 1:**
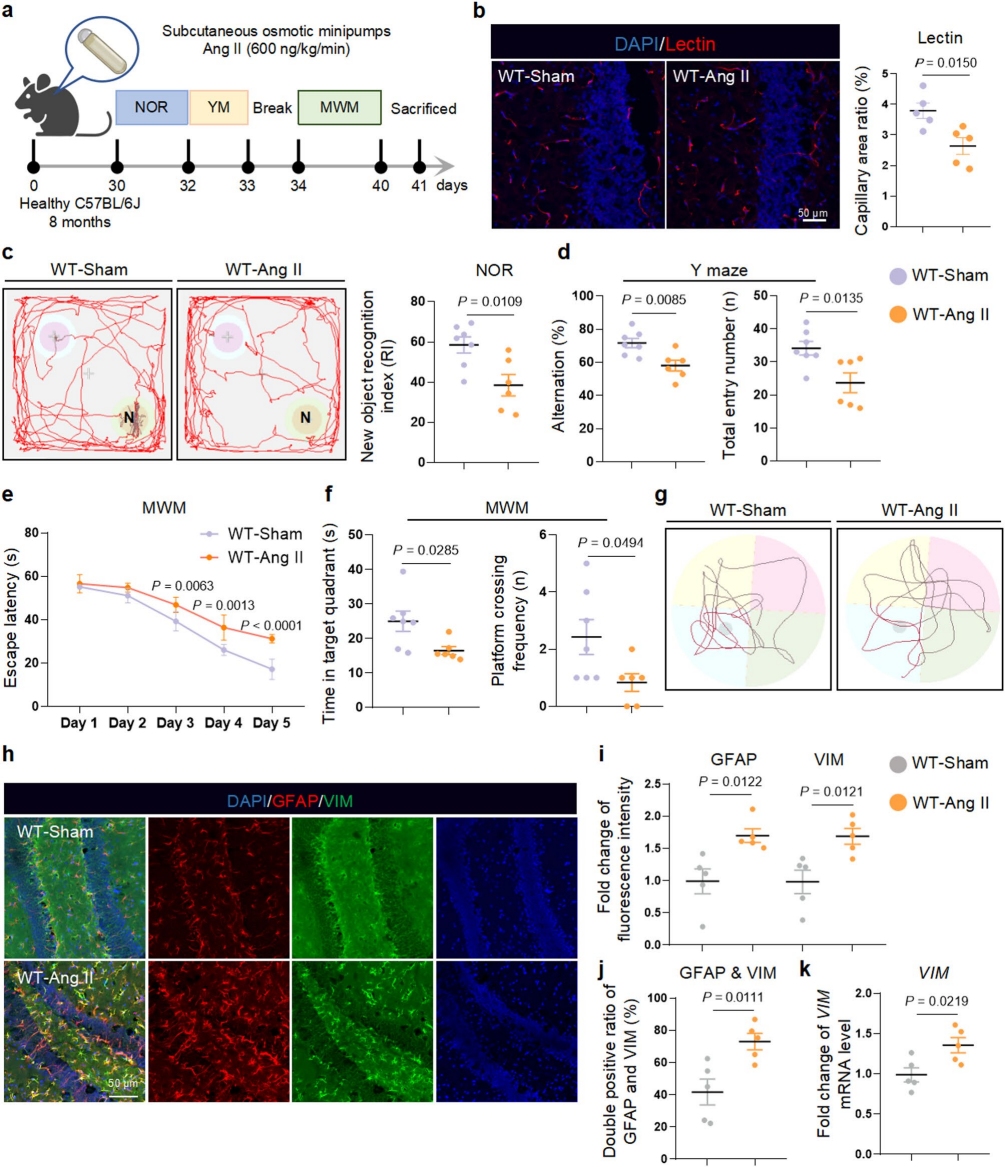
Vascular injury contributes to cognitive dysfunction and astrocyte reactivity. **a** Schematic illustration of the experimental timeline of WT-Sham and WT-Ang II mice. **b** Representative images and the quantification of capillary area ratio in the hippocampal dentate gyrus of WT-Sham and WT-Ang II mice (*n* = 5 mice per group). **c** Motion trails and the new object recognition index (RI) in new object recognition test (NOR). **d** The percentage of spontaneous alternations and total entry number in Y maze. **e-g** In Morris water maze test (MWM), the escape latency (**e**), the time in target quadrant, the platform crossing frequency (**f**) and the swimming track in the probe trial (**g**) (WT-Sham = 7 mice, WT-Ang II = 6 mice). **h-j** Representative images (**h**) and the quantification of GFAP and VIM (**i**,** j**) in the hippocampal dentate gyrus of WT-Sham and WT-Ang II mice (*n* = 5 mice per group). **k** mRNA levels of VIM in the hippocampus of WT-Sham and WT-Ang II mice (*n* = 5 mice per group). Data are shown as mean ± SEM, and the *P*-value was reported on the graph highlighted comparison by unpaired two-sided *t*-test (**b-d**,** f-k**) or two-way ANOVA with post-hoc Tukey adjustment (**e**)

To assess the effect of chronic vascular injury on astrocytes, we employed Angiotensin II (Ang II) to establish a mouse model of vascular injury (Fig. 1a) according to the method of *Faraco*,* G. et al.* [14]. A notably decreased capillary area ratio was observed in the dentate gyrus of the hippocampus after one-month of Ang II infusion, indicating the successful establishment of aberrant vascular injury model (Fig. 1b, Supplementary Fig. 1a, b). The vascular injury was accompanied by fibrosis, with a significant increase in the thickness of elastic fibers (Supplementary Fig. 1a, b). To be specific, vascular injury mice exhibited reduced recognition memory in the novel object recognition (NOR) task (Fig. 1c) and decreased spontaneous alternations in the Y maze task (Fig. 1d, Supplementary Fig. 1c). In the Morris water maze (MWM) task, these vascular injury mice showed an obviously prolonged escape latency to find the hidden platform in the training period (Fig. 1e). Meanwhile, the vascular injury mice manifested decreased resident time in the target quadrant and reduced platform crossing frequencies in the probe trials (Fig. 1f, g). Ang II treatment impaired the cognition of mice, but didn’t affect the moving speed and distance in all neurobehavioral tasks (Supplementary Fig. 1d).

We then conducted a glial neuropathological analysis to understand how vascular injury affected astrocytes and observed that hippocampal astrocytes were significantly activated in vascular injury mice (Fig. 1h, i). About 73% of the astrocytes expressed the reactive astrocyte biomarker VIM in Ang II-treated group (Fig. 1j, k). As a hydrophilic polypeptide with a relative molecular mass (Mr) over 1100, Ang II is a strong vascular regulator, mainly acting on vascular endothelial cells to accelerate endothelial cell aging and damage [15]. Moreover, Ang II is hard to cross the BBB [16] and the expression of Ang II receptors (AT1R and AT2R) is negligible in astrocytes (Supplementary Fig. 1e), therefore Ang II did not impact cell viability and astrocyte activation in vitro (Supplementary Fig. 1f-h). However, higher expression of vascular inflammatory factors (Supplementary Fig. 1i) and enhanced BBB permeability indicated by S100B were observed in the mice with Ang II treatment (Supplementary Fig. 1j). Notably, astrocyte endfoot marker AQP4 still maintained close connection with blood vessels in this model (Supplementary Fig. 1k), suggesting that the intense influence of Ang II on astrocyte reactivity excites an unrevealed underlying mechanism of BMECs and astrocytes interaction.

### CEEVs promote astrocyte reactivity within vascular injury

**Fig. 2:**
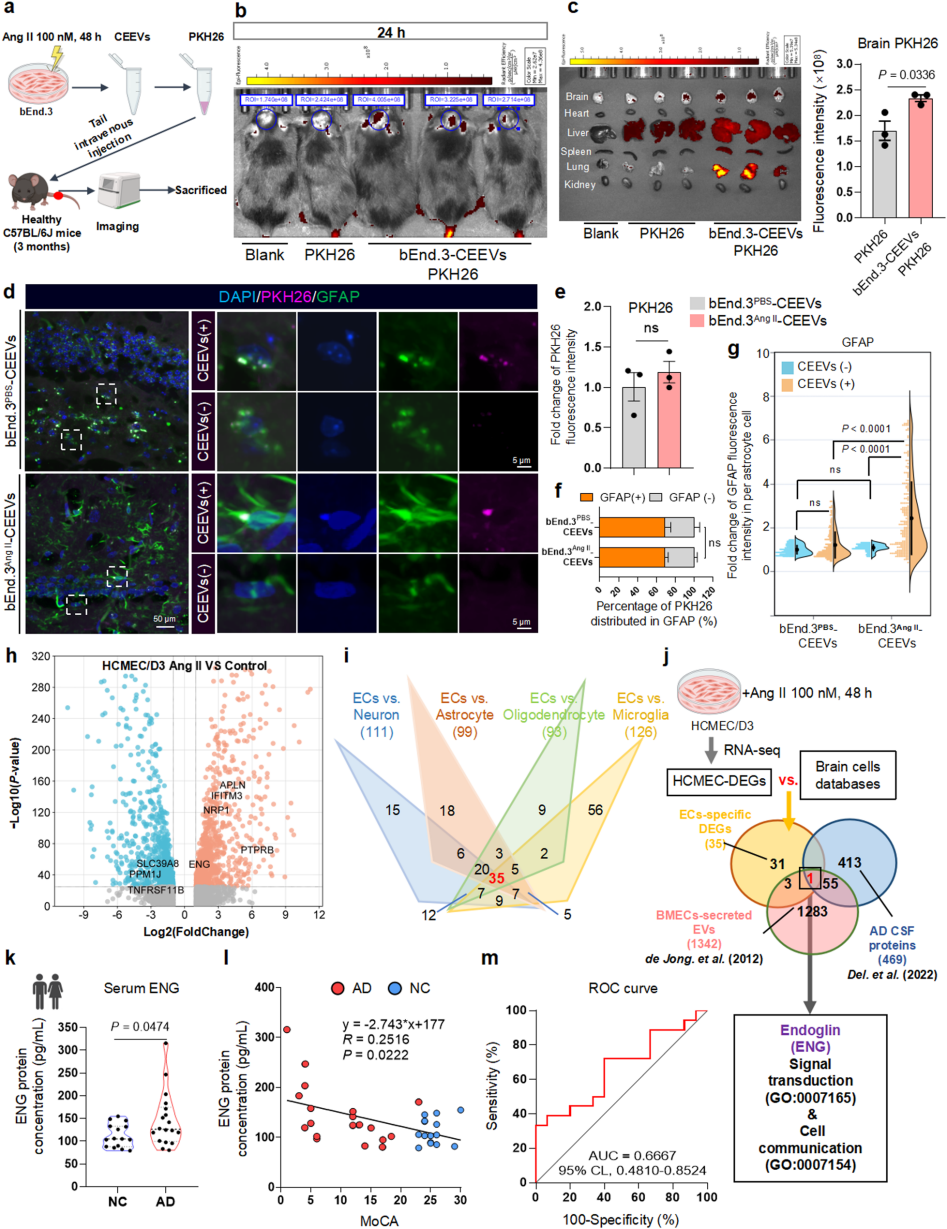
CEEVs specific endoglin (ENG) is a potential communicator between BMECs and astrocytes in AD. **a** Schematic and timeline of the transplantation treatment by PKH26-labeled cerebrovascular endothelial extracellular vesicles (CEEVs). **b**,** c** The bio-distribution of CEEVs-PKH26 in vivo at 24 h (**b**) and in body tissues 48 h after injection (*n* = 3 mice per group) (**c**). **d-g** Representative images (**d**), the quantification of PKH26 fluorescence intensity in hippocampal dentate gyrus (*n* = 3 mice per group) (**e**), the percentage of PKH26 distributed in GFAP staining (*n* = 3 mice per group) (**f**), and the quantification of GFAP per astrocyte cell with or without CEEVs from 3 mice per group (**g**). **h** Differential-gene expression analysis of HCMEC/D3 stimulated by Ang II. Dotted lines indicate HCMEC-DEGs cut-offs for |log2(fold change)|>1.5 and -log10 (*P*-value) of 1.3, corresponding to *P*-value < 0.05, 3 samples per group. **i** The number of specific genes of HCMEC-DEGs in endothelial cells compared with other cell types in brain (microglia, neuron, astrocyte and oligodendrocyte). **j** Venn diagrams showing the identification of ENG by intersecting the DEGs of injured BMECs from (**h**) with the differential proteome in the CSF of AD patients and the proteome of endothelial cell EVs from the datasets. **k-m** A cohort study of AD patients (*n* = 18) and non-demented control (*n* = 15). ENG protein levels were up-regulated in the serum of AD patients measured by ELISA (**k**). Scatter plot of Montreal Cognitive Assessment (MoCA) versus ENG levels was shown and the data were analyzed with a linear regression method (**l**) and ROC curve analysis of ENG (**m**). Data are shown as means ± SEM, and the *P*-value was reported on the graph highlighted comparison by unpaired two-sided *t*-test (**c**,** e**,** f**,** k**) and one-way ANOVA with post-hoc Tukey adjustment (**g**). ENG: Endoglin, ECs: endothelial cells, DEGs: differential genes, NC: Non-demented control

Considering Ang II triggering the release of cerebrovascular endothelial extracellular vesicles (CEEVs) from cerebral microvessels [17, 18] and the tight involvement of CEEVs in intercellular communication [4, 19, 20], we assumed that astrocyte reactivity induced by BMECs injury might result from the transmission of some key signal molecules via CEEVs. Therefore, we isolated CEEVs from Ang II-treated bEnd.3 cells, a mouse BMECs cell line, by ultra-high speed centrifugation (Supplementary Fig. 2a-c), and then transplanted the CEEVs into healthy mice by tail intravenous injection (Fig. 2a) according to the method of *Chen X*, et al. [21]. CEEVs possesses inherent homing abilities to the origin cells and BBB leapfrog ability [22, 23], and therefore the intravenous CEEVs showed intracerebral accumulation and BBB crossing in C57BL/6J mice (Fig. 2b, c). There was no significant difference in brain distribution of PKH26-labeled CEEVs isolated from PBS- or Ang II-treated bEnd3 cells (Fig. 2d, e), suggesting that Ang II treatment does not affect the entry of CEEVs into the brain. About 70% of CEEVs-PKH26 signals were colocalized with the astrocyte marker GFAP (Fig. 2d-f). Astrocytes cover more than 80% surface of vascular endothelial cells, indicating that they are the primary targets of vasogenic substances [24]. Our investigation specifically examined the impact of CEEVs on astrocytes and revealed that CEEVs from Ang II-treated bEnd.3 cells could cross BBB and successfully target astrocytes. Notably, CEEVs-positive astrocytes (CEEVs^+^) exhibited significantly higher activation levels compared to their CEEVs-negative counterparts (CEEVs^-^) (Fig. 2d, g), indicating that CEEVs may serve as molecular triggers initiating astrocyte reactivity. Since EVs exhibit little species specificity [25, 26], CEEVs from Ang II-treated HCMEC/D3, a human BMECs cell line, could also be effectively delivered to primary mice astrocytes (Supplementary Fig. 2d) and promoted astrocyte reactivity (Supplementary Fig. 2e). Taken together, these results indicated that injured BMECs could cause astrocyte reactivity through secreted CEEVs.

### Endoglin (ENG) in CEEVs is a critical mediator of BMEC-astrocyte crosstalk in AD

The CEEVs carry a variety of cargoes including proteins, lipids, DNAs and RNAs to facilitate local and remote communication among the cells of central nervous system (CNS) [27]. Recent studies have uncovered that the proteins derived from endothelial EVs are highly cell- and disease-specific and play an important role in neuroimmune communication [4, 19, 20]. To investigate whether proteins in CEEVs promote astrocyte reactivity, we incubated astrocytes with the CEEVs from injured BMECs pretreated with Triton X-100 and papain [28]. Results showed that CEEVs failed to induce astrocyte reactivity when proteins were degraded by Triton X-100 and papain (Supplementary Fig. 2f). All of these indicated that the astrocyte reactivity is predominantly mediated by the proteins in CEEVs.

To investigate the functional protein in CEEVs mediating the BMEC-astrocyte crosstalk, RNA-seq analysis was performed on Ang II-treated HCMEC/D3 to identify differential genes (DEGs), followed by a database mining strategy according to three inclusion criteria: (1) the candidate protein was specifically expressed in BMECs with a transcripts per million (TPM) value at least four times that of astrocytes and other brain cells (oligodendrocytes precursor cells, neurons, and microglia) (Supplementary Table 1, Fig. 2h, Supplementary Fig. 3a, b); (2) the candidate protein was specifically expressed in the EVs of human microvascular endothelial cells; (3) the candidate protein was detectable in the CSF of AD patients (Fig. 2i-j). We utilized transcriptional profiling analysis in a database of cell type expression categories [29] to screen the proteins higher expression in BMECs, and then examined their intersection with the EVs protein database [30] of human microvascular endothelial cells (Vesiclepedia_551 [31]), and the differential proteome in the CSF of AD patients [32]. Interestingly, only ENG was an overlapped DEG among these datasets (Fig. 2j) and the BMECs-specific expression pattern of ENG was validated by single-cell RNA-seq database (*Muris. et al.* [33]), qPCR, and Western Blotting (Supplementary Fig. 3c-e). Notably, ENG is highly conserved between human and mice, with a similarity up to 99% (Supplementary Fig. 4a). GO functional categorization revealed that ENG was involved in signal transduction (GO:0007165) and cell communication (GO:0007154) (Fig. 2j). Therefore, ENG might be a potential signaling mediator for BMEC-astrocyte crosstalk in AD.

Previous studies have reported that ENG was a biomarker of abnormal angiogenesis and increased in the serum and CSF of AD patients [32, 34, 35]. Consistent with these findings, we observed increased ENG in the hippocampus of APP/PS1 mice (Supplementary Fig. 3f) and in the serum of AD patients (Fig. 2k). The pathological upregulation of ENG in AD was not age or sex-dependent (Supplementary Fig. 3g, h). Furthermore, serum ENG levels showed a significant negative correlation with the Montreal Cognitive Assessment (MoCA) scores of AD patients and non-demented controls (Fig. 2l, Supplementary Fig. 3i). Receiver operating characteristic (ROC) curve analysis demonstrated the potential of serum ENG as a diagnostic biomarker, distinguishing AD patients from non-demented controls (AUC = 0.6667, 95% CI: 0.4810–0.8524; Fig. 2m). No significant differences in age or sex were observed between groups (Table 1, Supplementary Fig. 3g, Supplementary Table 2). Together, these findings reflected the crucial role of ENG in BMEC-astrocyte crosstalk and likely indicated serum ENG as a potential biomarker for AD.

**Table 1:** Patient demographics

		NC				AD				*P*-value
Number		15				18				N/A
Age		69.87 ± 3.62				72.33 ± 7.28				0.242
		51–60	61–70	71–80	80+	51–60	61–70	71–80	80+	N/A
		0 (0%)	9 (60.0%)	6 (40.0%)	0 (0%)	2 (11.1%)	3 (16.7%)	12 (66.7%)	1 (5.5%)	N/A
Sex	Male	6 (40%)				9 (50%)				0.566
	Female	9 (60%)				9 (50%)				
MoCA		25.33 ± 1.99				9.61 ± 6.17				< 0.0001****
ENG (pg/ml)		112.16 ± 25.77				146.86 ± 60.54				0.0474*

*NC: non-demented control, AD: alzheimer’s disease, ENG: endoglin, MoCA: montreal cognitive assessment. Categorical variables were described as n (%), and continuous variables were expressed as mean ± SD. N/A = not applicable. Continuous variables were assessed using independent samples student’s *t*-tests and categorical data were assessed using *χ*^2^ tests. *P < 0.05, ****P < 0.0001

### Injured BMECs deliver ENG protein to astrocytes through CEEVs

**Fig. 3:**
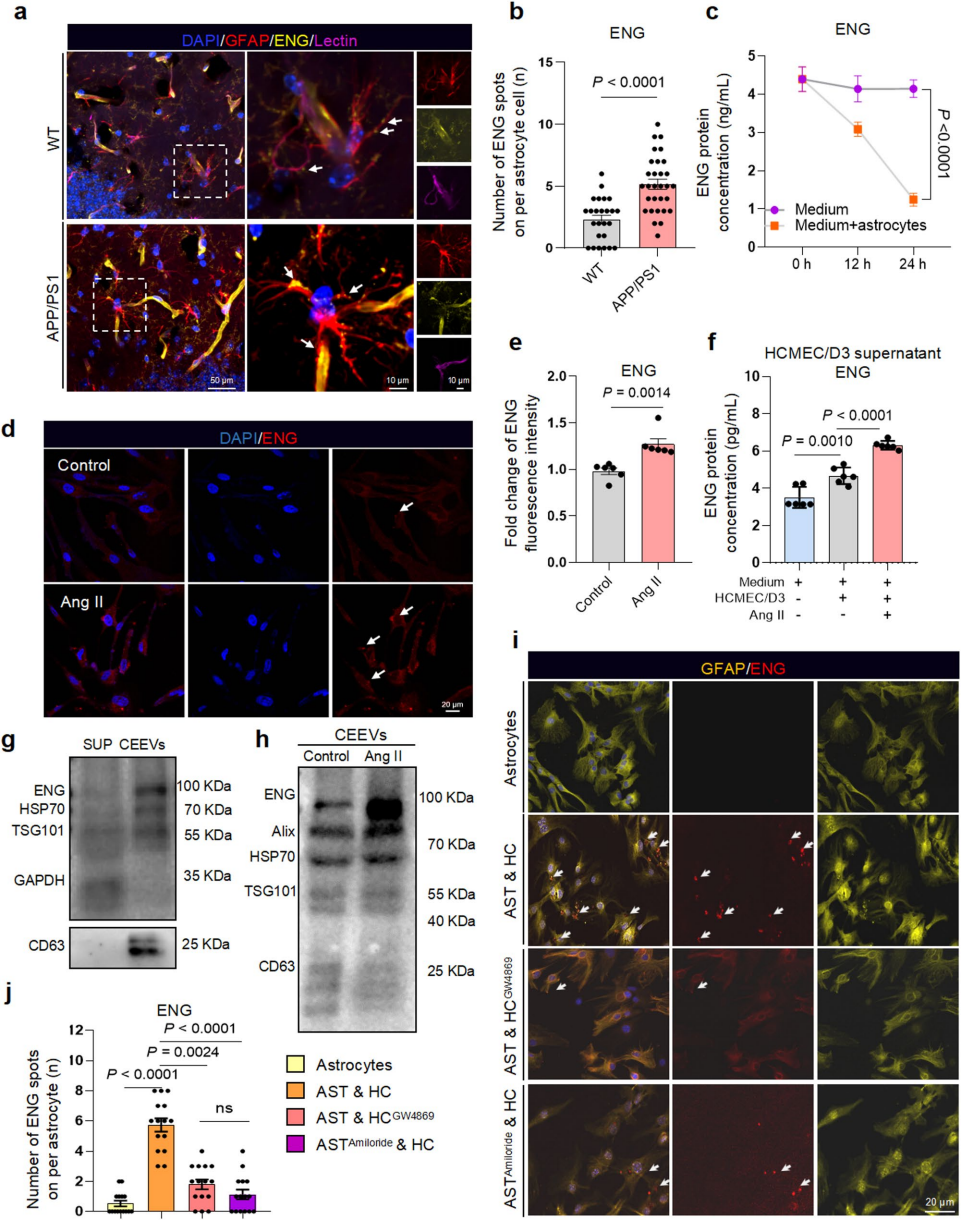
Injured BMECs deliver ENG protein to astrocytes through CEEVs. **a**,** b** Representative images (**a**) and the quantification (**b**) of ENG on per astrocyte in WT and APP/PS1 mice (*n* = 5 mice per group). **c** ENG protein levels in HCMEC/D3 conditioned-medium incubating with or without astrocytes (*n* = 6 biologically independent experiments). **d**,** e** Immunostaining (**d**) and the quantification (**e**) of ENG expression in HCMEC/D3 treated with Ang II (*n* = 6 biologically independent experiments). **f** ENG protein levels in HCMEC/D3 medium treated by Ang II (*n* = 6 biologically independent experiments). **g** Immunoblotting of distribution of ENG in HCMEC/D3 culture medium. **h** Protein content of ENG in the CEEVs from Ang II-treated HCMEC/D3 vs. normal control (*n* = 3 biologically independent experiments). **i**,** j** Representative images (**i**) and the quantification (**j**) of ENG spots on per astrocyte cell in AST & HC Co-culture system treated by GW4869 to HCMEC/D3 or Amiloride to astrocytes from 3 biologically independent experiments. Data are shown as means ± SEM, and the *P*-value was reported on the graph highlighted comparison by unpaired two-sided *t*-test (**b**,** e**), two-way ANOVA with post-hoc Tukey adjustment (**c**), one-way ANOVA with post-hoc Tukey adjustment (**f**) and Kruskal-Wallis with Dunn’s multiple comparisons test (**j**). AST: astrocytes, HC: HCMEC/D3

To verify whether ENG can be transmitted to astrocytes, we performed immunofluorescence staining and detected distinct ENG signals co-localizing with astrocytes adjacent to cerebrovascular regions in the hippocampal dentate gyrus (Fig. 3a). The point-like distribution of ENG on astrocytes was observed in both wild-type (WT) and APP/PS1 mice, and was more significant in APP/PS1 mice (Fig. 3a, b). An accelerated decline of ENG protein was observed in the conditioned medium of HCMEC/D3 co-incubated with astrocytes (Fig. 3c), further suggesting the transmission of ENG between cells. Considering that CEEVs from injured BMECs caused a stronger astrocyte reactivity, we investigated whether CEEVs from injured BMECs could deliver more ENG to astrocytes. Our results showed that Ang II treatment increased ENG expression in HCMEC/D3 (Supplementary Fig. 5a-c) and led to more spot-like distribution of ENG (Fig. 3d, e) with abundance of ENG in the cell medium (Fig. 3f). Notably, ENG secreted by BMECs was more likely to be enriched in CEEVs rather than present in a free form (Fig. 3g), which was consistent with the previous study [36]. Meanwhile, BMECs injury induced by other stimuli such as LPS, IL-6, and TNF-α analogously caused the expression and secretion of ENG (Supplementary Fig. 5d, e). Additionally, there was an increasing trend of ENG in the serum of aging mice and Ang II-induced vascular injury mice (Supplementary Fig. 5f, g). The ENG level in CEEVs was increased due to the BMECs injury (Fig. 3h) and the ENG-contained CEEVs could be effectively received by astrocytes (Supplementary Fig. 5h). These results suggested that vascular injury enhances ENG expression and release from BMECs via CEEVs.

**Fig. 4:**
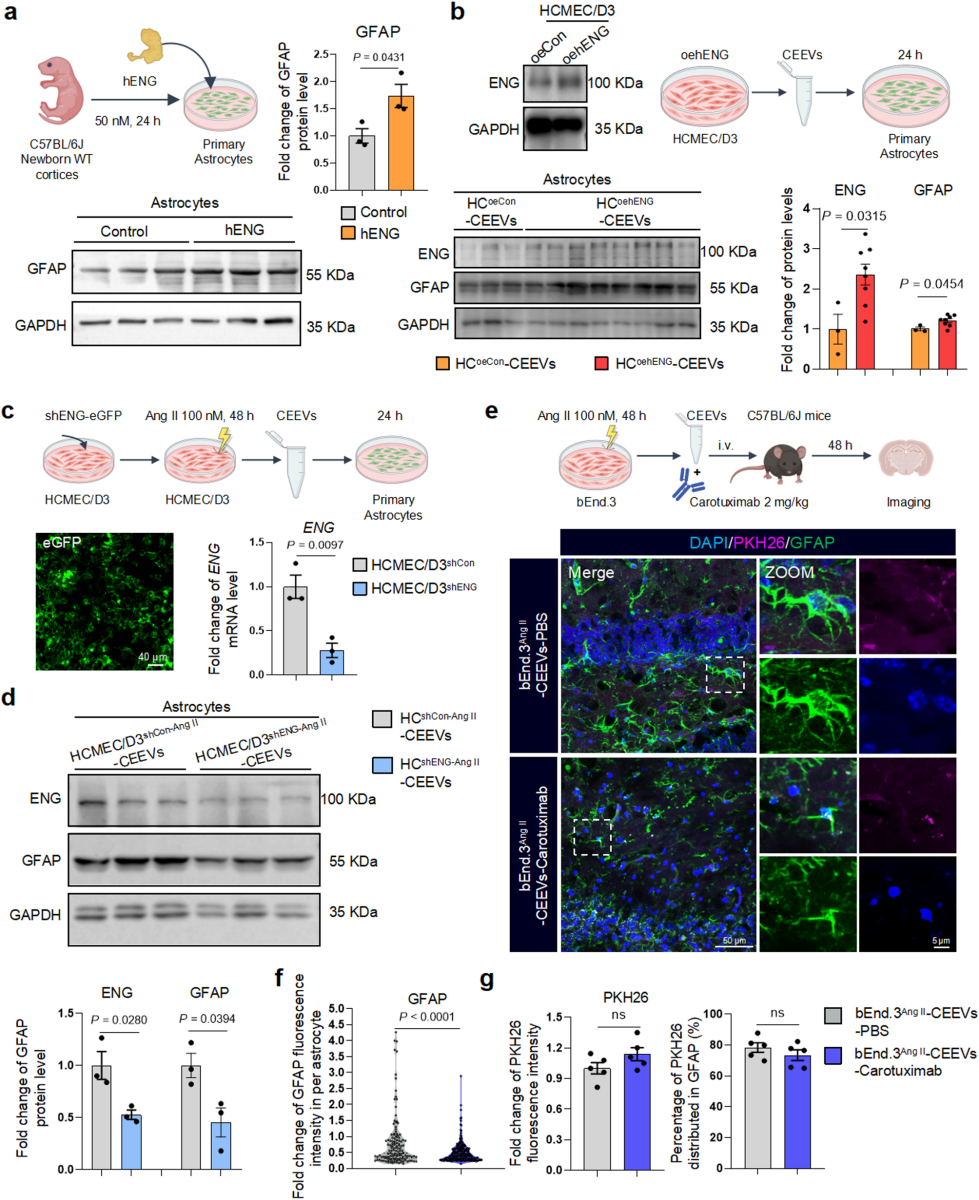
CEEVs switch astrocyte reactivity through ENG. **a** Immunoblotting and the quantification of GFAP in astrocytes treated by ENG recombinant protein (*n* = 3 biologically independent experiments). **b** Immunoblotting of GFAP in astrocytes treated by ENG-overexpressed CEEVs (*n* = 3/8 biologically independent experiments). **c**,** d** Knockdown of ENG in HCMEC/D3 and immunoblotting of ENG and GFAP in astrocytes treated by ENG-knockdown CEEVs (*n* = 3 biologically independent experiments). **e-g** Representative images (**e**) and the quantification of GFAP in per astrocyte (**f**) and PKH26 fluorescence intensity (**g**) in the hippocampus of CEEVs-treated C57BL/6J mice administrated with or without Carotuximab from 5 mice per group. Data are shown as means ± SEM, and the *P*-value was reported on the graph highlighted comparison by unpaired two-sided *t*-test

To determine the role of CEEVs-mediated ENG transmission between BMECs and astrocytes, we employed a transwell astrocyte and HCMEC/D3 co-culture system (Supplementary Fig. 5i). The GW4869, an EVs release inhibitor, was applied to HCMEC/D3 and Amiloride, a macropinocytosis inhibitor, was used to astrocytes in the co-culture system (Fig. 3i, j). Results indicated that inhibiting the release of CEEVs or engulfment by astrocytes significantly impeded ENG transmission from BMECs to astrocytes (Fig. 3i, j). Subsequently, to assess the impact of elevated ENG on astrocytes, we treated astrocytes with recombinant ENG protein, and observed increased astrocyte reactivity (Fig. 4a). This was confirmed by experiments showing that ENG overexpression in BMECs increased ENG delivery and astrocyte reactivity (Fig. 4b), while ENG knockdown in BMECs reduced ENG transfer and astrocyte activation (Fig. 4c, d). Furthermore, in C57BL/6J mice administered with ENG monoclonal antibody Carotuximab (TRC105) [37] while being concurrently exposed to CEEVs from Ang II-treated BMECs, we observed that Carotuximab attenuated CEEVs-induced astrocyte reactivity without affecting CEEVs’ intracerebral distribution or uptake by astrocytes (Fig. 4e-g). Taken together, these findings collectively demonstrated that elevated ENG levels within injured BMECs-derived CEEVs are crucial for triggering astrocyte activation.

### ENG promotes astrocyte reactivity and neuroinflammation via TGFBRI/Smad3 pathway

**Fig. 5:**
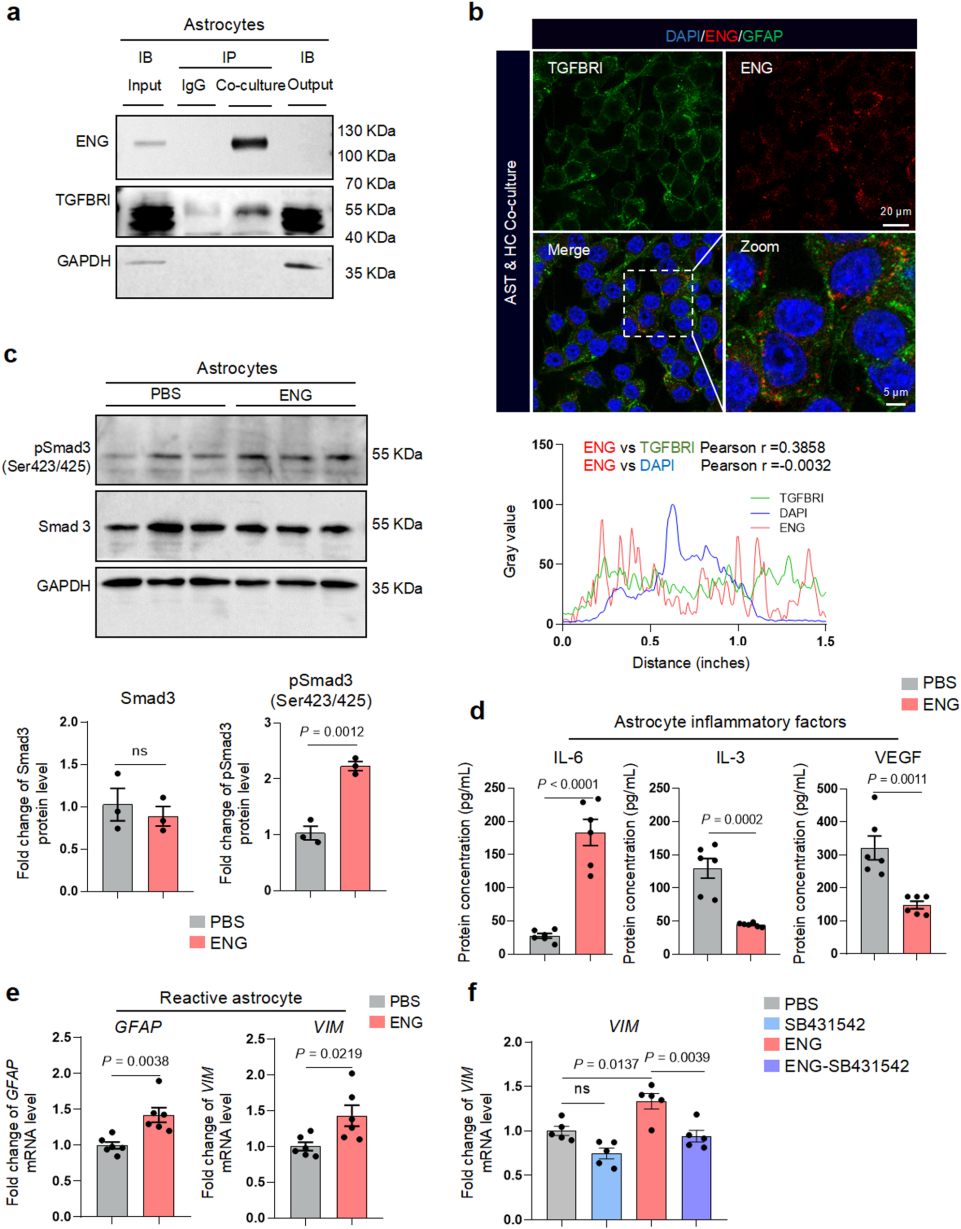
ENG regulates reactive astrocytes via TGFBRI/Smad3 pathway. **a** Immunoprecipitation and immunoblot analysis of ENG and TGFBRI in astrocytes of AST & HC Co-culture system. **b** Representative images and co-localization analysis of ENG and TGFBRI on astrocytes of AST & HC co-culture system. **c** Immunoblotting analysis and quantification of Smad3 and phospho-Smad3 (Ser423/425) in primary astrocytes treated with ENG recombinant protein (*n* = 3 biologically independent experiments). **d** The levels of IL-6, IL-3 and VEGF in the medium of ENG-treated astrocytes (*n* = 6 biologically independent experiments). **e** mRNA levels of *GFAP* and *VIM* in astrocytes treated by ENG (*n* = 6 biologically independent experiments). **f** Relative mRNA levels of *VIM* in astrocytes treated by ENG or SB431542 (*n* = 5 biologically independent experiments). Data are shown as means ± SEM, and the *P*-value was reported on the graph highlighted comparison by unpaired two-sided *t*-test (**c-e**) or one-way ANOVA with post-hoc Tukey adjustment (**f**)

Previous studies have reported that ENG is a pivotal accessory receptor of transforming growth factor β receptor (TGFBR) [37, 38] and plays an important role in strengthening the TGFBR/Smads signaling pathway (Supplementary Fig. 6a). TGFBR/Smad3 pathway is considered as an important pathway closely related to the dysfunction of astrocytes [39–41]. ENG delivered by CEEVs was mainly localized on the cell membranes of astrocytes (Supplementary Fig. 6b). However, only the TGFβ type I receptor (TGFBRI) was expressed on astrocytes, while TGFBRII was absent (Supplementary Fig. 6c, d). CO-IP and co-localization analysis further confirmed that BMECs-secreted ENG specifically bound to TGFBRI on astrocytes, rather than TGFBRII (Fig. 5a, b, Supplementary Fig. 6e). Smad3 is the downstream effector molecule of TGFβ signaling pathway to induce astrocyte activation [41, 42]. ENG promoted the phosphorylation of Smad3 without affecting the overall protein levels of Smad3 in astrocytes (Fig. 5c). The reactive astrocyte markers GFAP, VIM and IL-6 were all present in the ENG/TGFBR protein interaction network (Supplementary Fig. 6f). Consistently, ENG induced the release of astrocyte inflammatory factor IL-6, reduced the release of astrocyte functional molecules IL-3 and VEGF (Fig. 5d) and promoted astrocyte reactivity (Fig. 5e). Importantly, the TGFβ/Smad3 inhibitor SB431542 reversed the effect of ENG on astrocyte reactivity (Fig. 5f). Together, these results indicated that ENG secreted by BMECs interacts with TGFBRI on the astrocyte membranes, thus leading to astrocyte reactivity via TGFBRI/Smad3 signaling pathway.

**Fig. 6:**
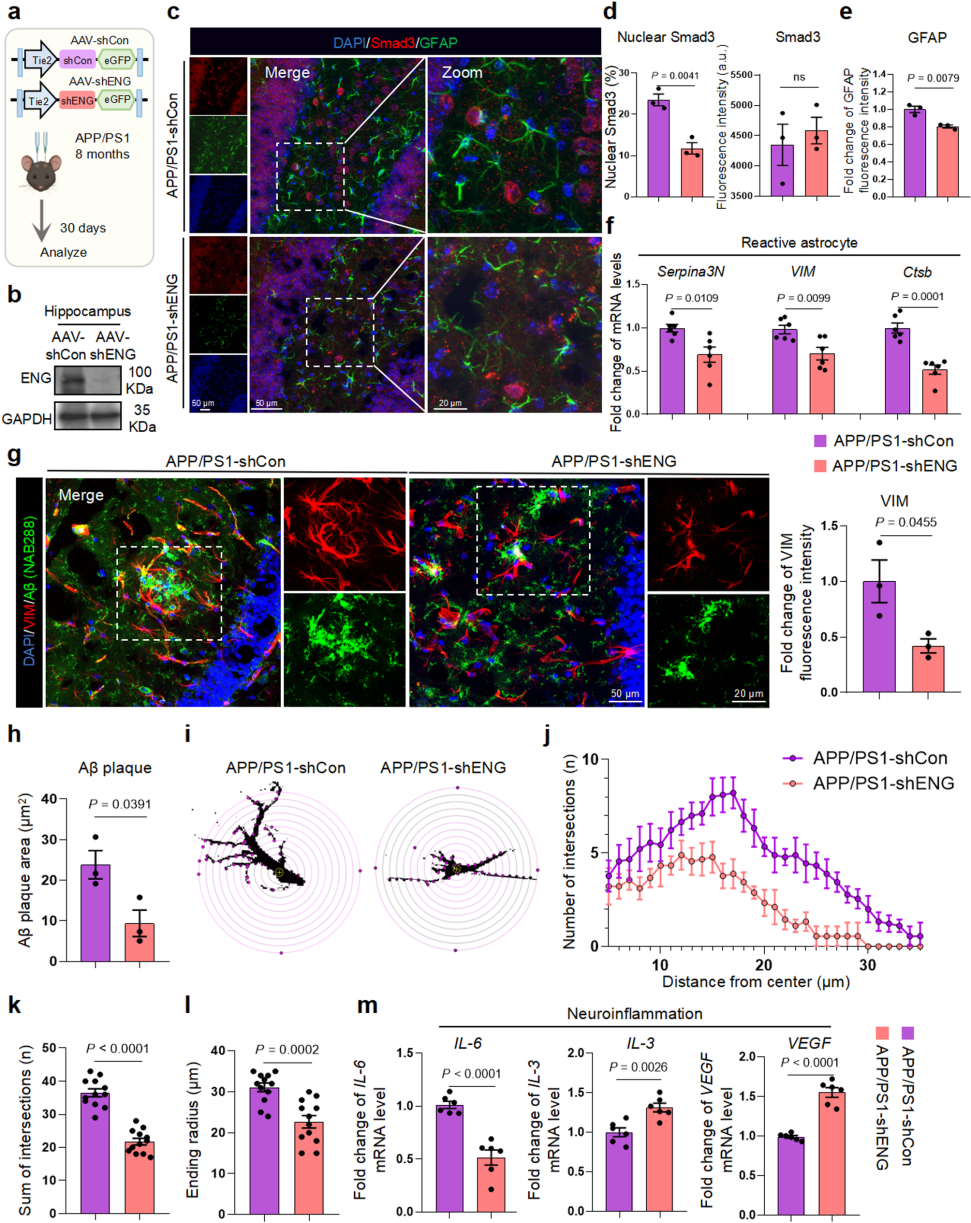
ENG deficiency inhibits astrocyte reactivity and neuroinflammation in APP/PS1. **a** Schematic of APP/PS1 experimental procedure of AAV-shENG interference. **b** Western blotting to identify ENG expression in the hippocampus of AAV-shENG injected APP/PS1 mice (*n* = 3 mice per group). **c-e** Representative images (**c**) and quantification of Smad3 (**d**) and GFAP (**e**) in the hippocampal dentate gyrus of APP/PS1-shCon and APP/PS1-shENG mice (*n* = 3 mice per group). **f** mRNA levels of *Serpina3N*, *VIM* and *Ctsb* in the hippocampus of APP/PS1-shCon and APP/PS1-shENG mice (*n* = 6 mice per group). **g**,** h** Representative images and quantification of VIM (**g**) and Aβ plaque (**h**) in the hippocampal dentate gyrus of APP/PS1-shCon and APP/PS1-shENG mice (*n* = 3 mice per group). **i** Sholl analysis of astrocytes from the VIM-stained images. Interval of concentric circles is 3 μm. **j** Average number of intersections in Sholl analysis with respect to the distance from the center of each VIM-stained cell. **k**,** l** The sum number of intersects (**k**) and the ending radius (**l**) by Sholl analysis of the stained VIM signal of each VIM-stained cell from 3 mice per group. **m** Relative mRNA levels of *IL-6*, *IL-3* and *VEGF* in the hippocampus of APP/PS1-shCon and APP/PS1-shENG mice (*n* = 6 mice per group). Data are shown as means ± SEM, and the *P*-value was reported on the graph highlighted comparison by unpaired two-tailed *t*-test

To further investigate the role of endothelial ENG downregulation in astrocyte reactivity in vivo, we engineered an adeno-associated virus serotype 9 (AAV9)-mediated delivery of ENG shRNA (Supplementary Table 3) under the endothelial cell-specific Tie2 promoter (AAV-shENG) in APP/PS1 mice. Following our previous method, AAV-shENG was injected stereotaxically into the hippocampus to specifically knockdown the expression of ENG in BMECs (Fig. 6a) [43]. Fluorescence analysis confirmed that the AAV-shENG-delivered eGFP signal localized primarily to microvessels (Supplementary Fig. 7a). AAV-shENG significantly reduced hippocampal ENG levels (Fig. 6b) and effectively normalized ENG expression to WT levels in APP/PS1 mice (Supplementary Fig. 7b, c). ENG downregulation did not alter the coverage of astrocyte endfoot AQP4 on BMECs, suggesting preserved structural integrity (Supplementary Fig. 7d). AAV-shENG effectively inhibited the transmission of ENG to astrocytes (Supplementary Fig. 8a, b) and blocked Smad3 nuclear translocation in astrocytes without altering total Smad3 (Fig. 6c, d), thereby inhibiting GFAP overexpression (Supplementary Fig. 8c) and astrocyte reactivity (Fig. 6e, f). GFAP displayed a significant positive correlation with the level of ENG in astrocytes (Supplementary Fig. 8d).

Additionally, Aβ plaque burden was significantly relieved alongside the reduced level of VIM in APP/PS1-shENG mice (Fig. 6g, h). VIM is a marker of reactive astrocytes and correlates with the ability of astrocytes to clear Aβ [44]. Sholl analysis revealed decreased astrocytic reactivity, branching and complexity shown as the number of branches, the sum of intersections, and the ending radius of astrocytes in APP/PS1-shENG mice (Fig. 6i-l). Furthermore, inflammatory factors secreted by astrocytes showed significant changes, with decreased *IL-6* and increased *IL-3* and *VEGF* expression in APP/PS1-shENG mice (Fig. 6m). Microglia activation was inhibited in mice with endothelial cell-specific ENG knockdown (Supplementary Fig. 8e-g). RNA-seq analysis demonstrated significant downregulation of the immune inflammatory response, including cytokine production, immune system process regulation, and leukocyte activation (Supplementary Fig. 8h, i). These results suggested that endothelial ENG downregulation suppresses astrocyte reactivity and neuroinflammation in APP/PS1 mice.

### Endothelium-specific knockdown ENG improves cognitive dysfunction in APP/PS1 mice

**Fig. 7:**
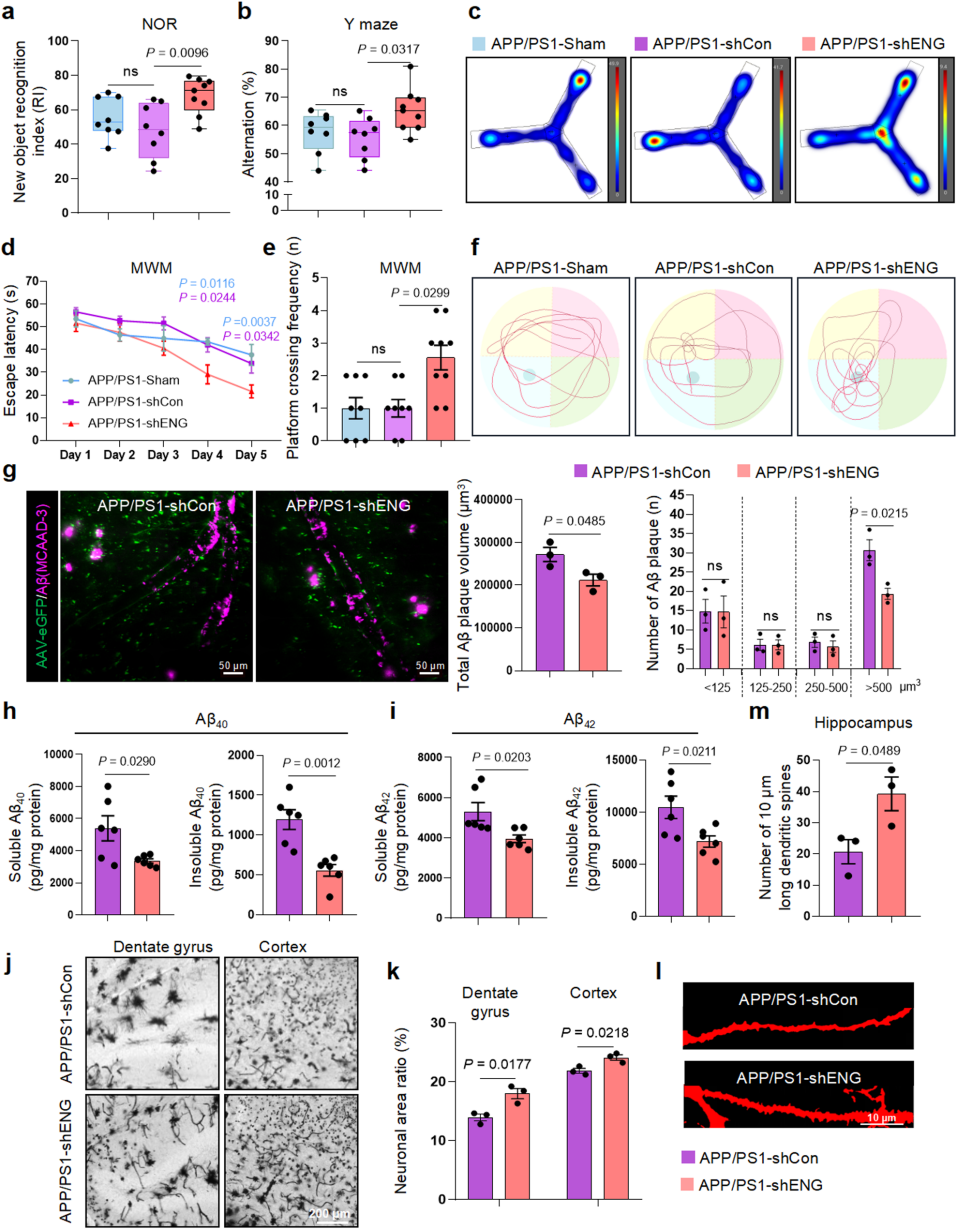
ENG deficiency in BMECs ameliorates cognitive disorder and AD pathology. **a** NOR test was performed on APP/PS1 mice one month after injection of PBS (APP/PS1-Sham, *n* = 8 mice per group), AAV-shCon (*n* = 8 mice per group) or AAV-shENG (*n* = 9 mice per group). **b**,** c** The percentage (**b**) and the heatmap (**c**) of spontaneous alternations in Y maze. **d-f** The escape latency during the learning task (**d**), the platform crossing frequency (**e**) and motion trails (**f**) in the probe trial test of MWM. **g** Two-photon analysis of Aβ plaques around blood vessels, the total volume of Aβ plaques around blood vessels and the number of Aβ plaques distributed in different volumes in APP/PS1-shCon and APP/PS1-shENG mice (*n* = 3 mice per group). **h**,** i** The protein level of soluble and insoluble Aβ_40_ (**h**) and Aβ_42_ (**i**) in the hippocampus of APP/PS1-shCon and APP/PS1-shENG mice (*n* = 6 mice per group). **j**,** k** Representative images (**j**) and quantification (**k**) of Golgi staining and neuron count in the dentate gyrus of APP/PS1-shCon and APP/PS1-shENG mice (*n* = 3 mice per group). **l**,** m** Spine density in the hippocampal dentate gyrus of APP/PS1-shCon and APP/PS1-shENG mice (*n* = 3 mice per group). Data are shown as means ± SEM, ns: non-significant, and the *P*-value was reported on the graph highlighted comparison by one-way ANOVA with post-hoc Tukey adjustment (**a**,** b**), two-way ANOVA with post-hoc Tukey adjustment (**d**), Kruskal-Wallis with Dunn’s multiple comparisons test (**e**) and unpaired two-sided *t*-test (**g-m**)

To investigate the in vivo effects of ENG on cognitive function, we investigated the effects of endothelial cell-specific ENG downregulation on cognitive performance and AD-associated neuropathologies in APP/PS1 mice. Showing no remarkable difference in body weight (Supplementary Fig. 9a), APP/PS1 mice with ENG knockdown (APP/PS1-shENG) exhibited significantly improvement of learning and memory, demonstrated by a 35% increase in NOR index (Fig. 7b, Supplementary Fig. 9b), a 20% increase in spontaneous alternation in the Y-maze test (Fig. 7c, Supplementary Fig. 9c, d), and a 30% reduction in escape latency in MWM training phase (Fig. 7d). During MWM probe trials, APP/PS1-shENG mice showed a significantly longer residence time in the target quadrant and a higher platform crossing frequency compared to control mice (APP/PS1-shCon) (Fig. 7e, f, Supplementary Fig. 9e). Movement velocities were similar across groups (Supplementary Fig. 9c-e). Endothelial cell-specific ENG knockdown did not affect cerebrovascular density, cerebrovascular segment number or length (Supplementary Fig. 9f), nor the expression of tight junction marker ZO-1 in the BMECs of APP/PS1 mice (Supplementary Fig. 9g). Importantly, we observed a significant reduction in large-size perivascular Aβ plaques (> 500 μm) (Fig. 7g), along with decreased soluble and insoluble Aβ_40_ and Aβ_42_ levels in the hippocampus of APP/PS1-shENG mice (Fig. 7h, i). Furthermore, ENG knockdown mitigated neuronal and synaptic loss (Fig. 7j, k), and restored neuronal dendritic spine density (Fig. 7l, m). These results highlighted ENG as a potential therapeutic target for AD.

**Fig. 8:**
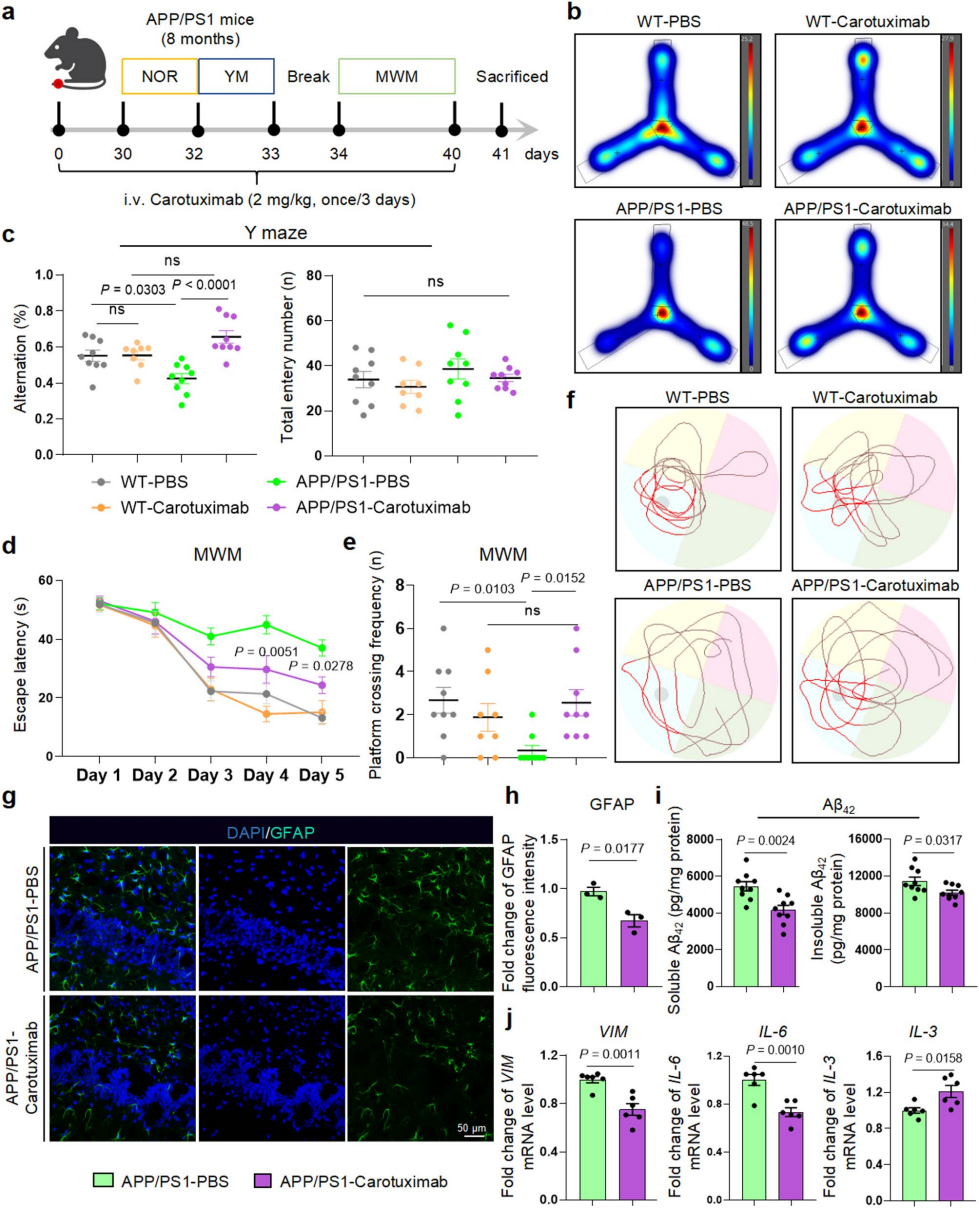
Carotuximab therapy improves the pathology of AD. **a** Schematic and timeline of Carotuximab administration and grouping, WT-PBS (*n* = 9 mice per group), WT-Carotuximab (*n* = 8 mice per group), APP/PS1-PBS (*n* = 9 mice per group) and APP/PS1-Carotuximab (*n* = 9 mice per group). **b**,** c** The representative heat maps of Y maze (**b**) and the percentage of spontaneous alternations in Y maze (**c**). **d-f** The escape latency during the learning task (**d**), the platform crossing frequency (**e**) and motion trail (**f**) in the probe trial test of MWM. **g**,** h** Representative images (**g**) and quantification (**h**) of GFAP in the hippocampal dentate gyrus of APP/PS1-PBS and APP/PS1-Carotuximab mice (*n* = 3 mice per group). **i** The protein level of soluble and insoluble Aβ_42_ in the hippocampus of APP/PS1-Sham and APP/PS1-Carotuximab mice (*n* = 9 mice per group). **j** mRNA levels of *VIM*, *IL-6*, and *IL-3* in the hippocampus of APP/PS1-shCon and APP/PS1-shENG mice (*n* = 6 mice per group). Data are shown as means ± SEM, ns: non-significant, and the *P*-value was reported on the graph highlighted comparison by two-way ANOVA with post-hoc Tukey adjustment (**c**,** d**), Kruskal-Wallis with Dunn’s multiple comparisons test (**e**) and unpaired two-tailed *t*-test (**h-j**)

Carotuximab is an ENG monoclonal antibody having demonstrated safety and efficacy in glioma treatment [45, 46]. After 40 days treatment with Carotuximab, APP/PS1 mice exhibited significant improvements in short-term memory, learning, and spatial memory (Fig. 8a-f), with no significant effect on recognition memory (Supplementary Fig. 10a). Carotuximab did not affect body weight or motor function (Supplementary Fig. 10b-d). Furthermore, Carotuximab treatment ameliorated several key neuropathological features, including astrocyte reactivity (Fig. 8g, h) and the high levels of soluble and insoluble Aβ_40_ and Aβ_42_ (Fig. 8i, Supplementary Fig. 10e). This was accompanied by decreased expression of *VIM* and *IL-6* and increased expression of *IL-3* (Fig. 8j). Altogether, these findings demonstrated that ENG antibody Carotuximab effectively mitigates neuropathological changes and improves cognitive function in APP/PS1 mice, suggesting its potential for the treatment of AD.

## Discussion

Cerebrovascular dysfunction plays a critical role in neurodegeneration [47], but the precise mechanisms linking vascular endothelial cell damage to cognitive decline in AD remain unclear. This study elucidated a novel mechanism underlying BMECs damage causing neurotoxic reactive astrocytes and cognitive impairment from the perspective of NVU. We demonstrated that ENG, delivered via CEEVs, is a key regulator of astrocyte reactivity through the TGFBRI/Smad3 pathway. Furthermore, ENG knockdown or functional inhibition by monoclonal antibody ameliorated astrocyte reactivity, improved synaptic function and cognition in AD model mice. Our findings suggest that targeting ENG may offer a potential therapeutic strategy for AD characterized by brain endothelial cell damage and astrocyte reactivity.

Recent large-scale clinical studies observed a direct correlation between untreated hypertension and accelerated cognitive dysfunction in AD [48]. Furthermore, a human brain vascular atlas showed that 30 of the top 45 genes associated with AD risk by genome-wide association studies (GWASs) were expressed in the human brain vasculature [49]. These clinical and single-nucleus transcriptomic studies together indicate that vascular impairment plays an important role in the early onset and progression of this most common neurodegenerative disease. Several hypotheses have been proposed to explain how neuronal function and cognition is impaired by brain vascular dysfunction upon AD pathology. For instance, BBB breakdown results in vasculotoxic and neurotoxic proteins entering the brain to cause neuronal injury, and cerebral blood flow reduction leads to a decrease in protein synthesis, which is indispensable for the synaptic plasticity mediating learning and memory [50]. Recently, transcriptomic researches made comprehensive characterization on brain vasculature and highlighted the dysregulation of intercellular communication in AD [2, 51, 52]. Since AD is a complex pathophysiologic process involving multiple cell types, our current study revealed BMEC-astrocyte signal transduction in NVU and provide evidences for a central role of brain endothelial cells to drive cognitive decline in AD.

Previous studies have suggested that reactive astrocytes were highly present in the brain samples of AD patients, and directly participated in Aβ clearance, tau pathology, microglia regulation and neurodegeneration [8, 9]. In NVU, much attention has been put on the effects of astrocytes on vascular function such as repairing and remodeling [53–55], while fewer studies have focused on the role of vascular-derived molecules on astrocytic function. It is encouraging that the potential of endothelial cells as active regulators of astrocytic function upon various physiopathological conditions has been gradually recognized. Endothelial cells have been reported to modulate astrocytes to neural progenitor cell trans-differentiation through releasing microvesicles with pro-neural factor Ascl1 in a mouse model of stroke [4]. However, whether endothelial cells can regulate astrocyte reactivity in the pathological process of AD is still obscure. We for the first time showed that CEEVs from BMECs could act as an intercellular inducer of astrocyte reactivity, providing a working model for BMECs-mediated astrocytic regulation upon vascular injury.

CEEVs are a heterogeneous group of cell-derived membranous structures which are now regarded as an indispensable mechanism for intercellular communication by exchanging DNA/RNAs, proteins and metabolites among cells [56]. Direct communication between brain blood vessels and nervous system mediated by CEEVs represents a significant signaling transmission in neurovascular interactions in CNS. Previous studies have reported that the release of CEEVs was enhanced and their composition was altered in both MCI and AD [57]. AD-brain-derived extracellular vesicles could impair cognitive function in mice [58]. However, the specific mechanism of CEEVs participation in AD remains to be elucidated. The hunting for active molecules within CEEVs provides an opportunity to comprehensively delineate the functions of vascular injury on neurodegenerative process.

Here by integrating transcriptomics and proteomics data, we identified ENG as a main regulator that accounts for the impact of CEEVs on adjacent astrocyte reactivity through binding to TGFBRI receptor on astrocytes and activating TGFβ/TGFBRI/Smads pathway. Importantly, even though astrocytes are spatially close to BMECs in NVU, we cannot exclude the possibility that endothelial cells may influence neuronal function through other regulator factors in CEEVs. In addition, since ENG has been shown to be up-regulated in various vascular pathologies, including hypertension, stroke and diabetes [59–61], it is of great significance to further explore the upstream regulators of elevated ENG expression in AD.

In the CNS, TGFβ is primarily produced by astrocytes and mainly regulates neuronal cell growth, angiogenesis, and microglia maturation [62, 63]. However, due to the low expression of TGFBRII in astrocytes under physiological conditions, the direct influence of TGFβ on astrocytes is relatively weak. Nevertheless, in pathological process such as AD, TGFβ could promote astrocyte reactivity, up-regulate IFITM3 expression, activate γ-secretase and promote Aβ accumulation through TGFBRI/Smad signal pathway [39, 40, 64], suggesting that astrocytes could be activated by TGFβ with the assistance of ENG. To the best of our knowledge, this is the first study demonstrating ENG as a switch of astrocyte reactivity in the pathological regulation of AD.

Our present study evaluated the feasibility of using ENG as a therapeutic target for AD and reported for the first time that specifically decreasing the expression of ENG in BMECs or systemic administration of an ENG antibody Carotuximab can improve cognitive dysfunction caused by vascular injuries. This therapeutic approach holds promising clinical significance, since studies have proved that Carotuximab is safe and effective in the clinical treatment of glioma [45, 46] and blocking TGFβ pathway in astrocytes holds promise for treating AD [62, 65].

Although our experimental findings demonstrate the preliminary efficacy of ENG-targeted interventions in preclinical AD models, several critical dimensions require further exploration. It should be noted that long-term ENG suppression may disrupt vascular homeostasis [66], and administration of Carotuximab could potentially elicit adverse events such as fatigue and headache [67]. The optimization of therapeutic agents demands enhanced pharmacokinetic stability and safety profiles. In addition, the implementation of personalized therapeutic strategies integrated with precision medicine platforms that account for age-specific, sex-dependent, and disease-stage-specific pathophysiological profiles will be critical for establishing ENG as a viable molecular target in AD management.

## Conclusion

We demonstrate a novel mechanism underlying the contribution of cerebrovascular dysfunction to AD pathogenesis. ENG is overexpressed under the conditions of BMECs injury and AD, and can be delivered to adjacent astrocytes via CEEVs to mediate astrocyte reactivity. Endothelial cell-specific ENG knockdown or ENG antibody treatment significantly reduces reactive astrocytes and improves cognitive performance of APP/PS1 mice. These findings establish a causal link between ENG-mediated BMEC-astrocyte crosstalk and AD pathogenesis and represent a potential avenue for developing disease-modifying therapies for AD.

## Materials and methods

### Human serum samples and study approval

The research protocol was approved by the ethics committee at Shanghai Ruijin Hospital, Shanghai Jiao Tong University School of Medicine and participants or their legally authorized representatives provided written informed consent. Patients with AD (*n* = 18) were enrolled through Shanghai Ruijin Hospital. Non-demented control subjects (*n* = 15) were recruited through network publicity and community outreach of Sheshan, Shanghai. Detailed information of patients and non-demented control is presented in Table 1. All AD patients had no evidence of other neurological diseases based on neuropathological examination.

### Mice and ethics statement

Healthy 8-month-old male wild type (WT) and transgenic mouse line overexpressing hAPP695swe (APP) and presenilin-1M146V (PS1) mutations (APP/PS1) C57BL/6J mice were purchased from the Cavens Biogle Model Animal Research Co.Ltd (Suzhou, Jiangsu, China). Mice were housed 4–5 mice per cage at a constant temperature (24 ± 2 ℃) with ad libitum access to food and water on a 12 h light/dark cycle. The numbers of animals used in each experiment are shown in the corresponding figure legends. All procedures were performed in accordance with the Ethics Committee of Shanghai Jiao Tong University School of Medicine. Investigators were blinded to the group allocation.

### Mice treatment

#### Stereotactic injection

To knock down ENG in the BMECs of APP/PS1 mice, the mice were anesthetized by inhalation of 2.5% isoflurane and positioned in a stereotaxic apparatus. According to our published method [43], the adeno-associated virus carrying either the control shRNA (AAV-shCon) or the ENG shRNA with Tie2 promoter and eGFP (AAV-shENG) (4 µL, 1.2 × 10^13^ viral genomes/mL, Genomeditech Co.Ltd, Shanghai, China) was microinjected into the hippocampus of mice using the following microinjection coordinates: anteroposterior − 2.06 mm, lateral ± 1.52 mm, and ventral − 2.2 mm. The sham operation group was injected with the same dose of PBS. Behavioral assays were performed one month later. The mice were sacrificed by rapid cervical dislocation under isoflurane anesthesia and the organs were dissected for further analysis. The sequence of the AAV-shENG and AAV-shCon for injection were shown in Supplementary Table 3.

#### Osmotic minipump implantation

According to *Faraco*, et al. [14]., Micro-Osmotic pumps (ALZET, #MODEL1004, USA) containing vehicle (0.9%, saline) (WT-Sham) or Ang II (600 ng/kg/min, APExBIO, #A1042, USA) (WT-Ang II) were implanted s.c. in C57BL/6J mice for 28 days to induce vascular injury.

#### Carotuximab treatment

According to the treatment strategy of *Dourado*, et al. [37]., Carotuximab (TRC105, 2 mg/kg) (MCE, #HY-P99494, USA) was administered i.v. every 3 days for a month. The sham group was given the same amount of PBS every 3 days.

### Drug treatment

HCMEC/D3 or bEnd.3 cells were treated with Ang II at a concentration of 100 nM for 48 h for immunofluorescence, qRT-PCR or Western blotting. HCMEC/D3 cells were treated with LPS (Sigma-Aldrich, #L2630, USA) at a concentration of 100 ng/ml, IL-6 (SinoBiological, #GMP-10395-HNAE, China) at 10 ng/mL or TNF-α (SinoBiological, #10602-HNAE, China) at 50 ng/mL for 24 h for qRT-PCR and ELISA test. Mice primary astrocytes were treated with recombinant human ENG protein (SinoBiological, #10149-H02H, China) at 50 ng/mL or SB431542 (MedChemExpress, #HY-10431, USA) at 10 µM for 24 h for qRT-PCR or Western blotting. All reagents were listed in Supplementary Table 4.

### Primary astrocytes isolation and culture

Primary astrocytes were prepared from C57BL/6J newborn pups within 24 h of birth as *Sanchez-Mico*,* M. V. et al.* [8] previously described. Briefly, the hippocampus tissues were isolated from the newborn pups (P0-P1) and cut finely in Hank’s balanced salt solution (HBSS) dissection media. Next, tissues were digested with 2.5% trypsin for 5 min at 37 ℃ and the treatment was stopped using complete DMEM with 10% FBS and the cells were mechanically dissociated. Then, the debris was eliminated by filtration (40 μm; BD Falcon) followed by centrifugation for 5 min at 1500 rpm. Afterward, the mixed glia cells were cultured in T75 flasks in complete media (DMEM with 10% FBS, 2 mM L-glutamine and 1% (v/v) penicillin/streptomycin) for 12–14 days. Mixed glia cultures were allowed to grow for one week, then microglia were separated by shaking the confluent flask at 250 rpm for 2.5 h twice with an interval of 48 h. Astrocytes were seeded on poly-D-lysine coated plates and experiments were performed 24–48 h after seeding.

### Astrocytes and BMECs co-culture system

To explore intercellular communication between BMECs and astrocytes, an in vitro coculturing system was established with HCMEC/D3 cells and primary astrocytes inoculated respectively in the upper and lower chambers of Corning^®^ Transwell^®^ (Corning, #3460, USA) cell culture insert with a polyethylene glycol terephthalate membrane (0.4 μm pore, Corning Inc., USA). In the Transwell co-culture experiment, HCMEC/D3 cells were pretreated with 20 µM GW4869 (MedMol, #S81516, China) for 30 min, and the primary astrocytes were pretreated with 15 µM Amiloride (MedChemExpress, #HY-B0285, USA) for 30 min, respectively. After the cells were washed twice with PBS (Thermo fisher scientific, #70011051, USA), the two kinds of cells were co-cultured in Transwell for 12 h and then observed by immunofluorescence.

### Extracellular vesicles (EVs) isolation and purification

HCMEM/D3 cells were cultured in medium for three passages and then seeded in T75 flasks. Cells were maintained until 80% confluence, then replaced the medium with serum-free medium and cultured for 24 h. EVs were firstly enriched by Meilunbio EVs isolation kit (Meilunbio, #MA0402, China) from the collected supernatants, and then isolated by ultra-high-speed centrifugation. In brief, a successive centrifugation at speeds of 300 *g*, 2,000 *g* and 10,000 *g* was adopted to eliminate the large dead cells and cell debris. Each step was at 4 °C for 10 to 60 min. Then the final supernatant was ultracentrifuged at 110,000 *g*, 4 °C for 90 min. Subsequently, the pellet was washed in 25 mL PBS, and recentrifuged at 110,000 *g*, 4 °C for 90 min. The final pellet was purified EVs and was resuspended in PBS pH7.4 and stored at -80 ℃. EVs was validated by western blot analysis and transmission electron microscope (G2 spititi FEI, Tecnai). The concentration of EVs was detected by Nanoparticle Tracking Analysis (Malvern, NanoSight NS300, UK).

### EVs transplantation and co-culture

In vivo experiments, EVs were isolated from the bEnd.3 cell medium and subsequently diluted in PBS to achieve a concentration of 1.80 µg of total protein per microliter imitated Chen et al. [21]. Healthy male mice (3 months) were tail intravenously injected with 200 µL of EVs from injured bEnd.3 (Ang II 100 nM, 48 h). At 24 h and 48 h timepoints, the distribution of EVs in mice was evaluated, then the brain slices were prepared. In vitro experiments, EVs (50 µg of total protein content suspended by PBS) were isolated from injured HCMEC/D3 cell medium following the above protocols and then these EVs were incubated with 1 × 10^6^ primary astrocytes and cultured in a humidified atmosphere with 5% CO_2_ at 37 °C. After 24 h, total protein was extracted. As a control, the EVs from normal HCMEC/D3 were simultaneously monitored.

### EVs labeling and tracking

EVs were labeled by PKH26 Red Fluorescent Cell Linker Kit (Sigma-Aldrich, #PKH26GL, USA) according to our previous methods [22]. In brief, EVs diluted in PBS were mixed with PKH26 dye (1 µL PKH26 in 200 µL diluent C) for 5 min, added 400 µL 1% BSA for 1 min and ultracentrifugated at 110,000 *g* for 90 min. Then the precipitation was retained and the pellet was resuspended in PBS. The labeled EVs were intravenously injected into healthy C57BL/6J mice, and the EVs distribution was observed at 24 h and 48 h after administration. Subsequently, the mice were anesthetized and transcardially perfused with ice-cold PBS to eliminate any remaining blood-derived EVs in tissues. Mouse brain slices were excised and traced for red fluorescence signals of EVs under confocal microscopy (Nikon, ECLIPSE Ti2, Japan).

### *In vivo* real-time imaging and biodistribution of CEEVs

The biodistribution of CEEVs was investigated in C57BL/6J mice using an in vivo imaging system (IVIS Spectrum, PerkinElmer). The excitation wavelength used was 551 nm, and the emission wavelength was 567 nm with PKH26 as the fluorescent probe. PBS was injected at the same volume (200 µL) as a blank and unlabeled PKH26 dye solution was injected as a control.

### Protein extraction and immunoblotting

Mouse tissues or cell pellets were lysed and homogenized in RIPA buffer with protease and phosphatase inhibitors (Beyotime, #P0013B, China), and immunoblotting was carried out according to our previous procedures (*Song*,* C. et al.* [68]). The antibodies used for immunoblotting in this study were listed in Supplementary Tables 4 and shown as follows: GFAP (Abcam, #ab4648, 1:1000), ENG (Abcam, #ab221675, 1:1000), TGFBRI (Abcam, #ab235578, 1:1000), TGFBRII (Abcam, #ab269279, 1:1000), Smad3 (CST, #C67H9, 1:1000), Phospho-Smad3 (Ser423/425) (CST, #C25A9, 1:1000), GAPDH (Abcam, #ab181602, 1:1000), CD63 (Abcam, #ab134045, 1:1000), Alix (Abcam, #ab275377, 1:1000), TSG101 (Abcam, #ab125011, 1:1000), HSP70 (Abcam, #ab51052, 1:1000).

### RNA isolation, reverse transcription and RT-qPCR

Total RNA was extracted from cells and mouse tissues using a SteadyPure Universal RNA Extraction Kit (Accurate Biotechnology Co., Ltd, #BSC52M1, China). Reverse transcription was carried out on the isolated RNA using a PrimeScript 1st Strand cDNA Synthesis Kit (TaKaRa Biotechnology, #RR036A, Japan) according to the manufacturer’s instructions. The concentration of nucleic acid was measured by ultramicrospectrophotometer at the wave length of 260 nm (Thermo, NANODrop 2000 C, USA). The RT-qPCR analyses were performed using TB Green Premix Ex Taq (TaKaRa Biotechnology, #RR420A, Japan) and on a LightCycler480 System (Roche, Switzerland). Housekeeping gene GAPDH was used as a control. Relative levels of gene expression were quantified by the Bio-Rad CFX manager. The primer sequences were shown in Supplementary Table 5.

### Immunofluorescence

Immunofluorescence was performed as our previous procedures (*Song*,* C. et al.* [68]). Briefly, mouse hippocampus samples were fixed in 4% paraformaldehyde and dehydrated in 20% and 30% sucrose in PBS and then microtome-cut into 20 μm thick sections on a cryostat. Cells were washed with PBS three times for 5 min each time and fixed in 4% paraformaldehyde for 15 min at room temperature. The sections or cells were blocked with Blocking Buffer for Immunol Staining (Beyotime, China) at room temperature for 2 h, and then incubated with the primary antibody as follows: GFAP (CST, #3670, 1:500), mouse anti-ENG (Abcam, #ab156756, 1:500), rabbit anti-ENG (Abcam, #ab221675, 1:1000), TGFBRI (Abcam, #ab235578, 1:500), Smad3 (CST, #C67H9, 1:500), APP/β-Amyloid (CST, #2450S, 1:500), Iba1 (Abcam, #ab178847, 1:500), VIM (Abcam, #ab194719, 1:500), AQP4 (Abcam, #ab259318, 1:500), ZO-1 (Abcam, #ab96587, 1:500). Next, appropriate secondary antibodies (Abcam, Alexa Fluor 555 or 647) were used followed by incubation with DAPI.

The fluorescence images were taken on a Nikon Ti2 microscope (ECLIPSE Ti2, Nikon, Japan) with confocal microscope at 40 × oil objective (1.3 numerical aperture) with a 2 × zoom for 20–30 μm total stacks with each step of the z-plane being 0.5 μm using NIS Elements software. Images were analyzed by Nikon Elements General Analysis tool for GFAP^+^ astrocytes and VIM signal (where at least 1 pixel of GFAP signal contacts 1 pixel of VIM signal). This tool was also used for PKH26 and Aβ plaque signal analysis. Mean Aβ plaque size was calculated by dividing Aβ plaque area signal by number of Aβ plaques detected. The PKH26 signal where at least 50% PKH26 signal overlaps with GFAP was considered to be taken up by astrocytes. Confocal images were acquired and analyzed in a blinded manner and the quantitative analysis was performed in the dentate gyrus of hippocampus unless otherwise mentioned.

### Sholl analysis

Sholl analysis was executed on sequentially layered and maximally projected confocal images as previously delineated (*Ju et al.* [69]). Immunofluorescence images of brain sections immunostained with VIM antibody were utilized for Sholl analysis. The Sholl analysis plugin applied in ImageJ (NIH) constructed a series of concentric circles at 3 μm intervals, originating from the center of the VIM signal (soma) and extending to the terminal point of the farthest process of each astrocyte. The quantity of VIM-positive process intercepts at each circle and the radius of the most extensive circle intersecting the astrocytes were examined and reported.

### RNA sequencing

High-throughput RNA sequencing analysis was conducted using the double-ended sequencing mode of illumina Hiseq sequencing platform by the Xurangene Company (Shanghai, China). Following normalization, log2 transformation, probe annotation, and differentially expressed genes (DEGs) were identified based on the criteria of |log2 fold change|>1 and adjusted *P* value < 0.05. Hub DEGs were selected for further investigation.

### Co-immunoprecipitation

Astrocytes from the AST & HC CO-culture system were lysed in Pierce™ IP lysis buffer (Thermo Fisher Scientific, #87787, USA) with a protease inhibitor cocktail (APExBIO, #K1007, USA). Anti-ENG antibody (Abcam, #ab231774, 1:50) was added to the obtained samples and then incubated at 4 °C overnight. The lysates were incubated with Pierce™ Classic Magnetic Bead IP/Co-IP Kit (Thermo Fisher Scientific, #K1007, USA) on a rotator for 1.5 h. Finally, proteins were eluted from the resin with 2×SDS sample buffer and used for immunoblotting analysis.

### Behavioral assays

Group-housed male mice were used for behavioral tasks to avoid estrogen-related and other cognition-irrelevant variations [70, 71]. One month after microinjection of AAV-shCon or AAV-shENG, mice underwent behavioral testing. Mice were habituated to the testing room 1 h prior to each assay.

#### Novel object recognition (NOR)

Before the experiment, mice were placed into the open field (60 cm long and 60 cm wide with 60 cm walls) for 10 min. Then two identical objects were positioned in the open field and each mouse was allowed to explore the objects freely for 5 min. After 24 h, one of the two objects was replaced by a new object and each mouse had the same time to explore the objects. After each exploration, the chamber was wiped with 70% ethanol. The preference of each mouse for the two objects was recorded and quantified by a video tracking software (Noldus Tech, EthoVision XT 16, Netherlands).

#### Y-maze spontaneous alternation

The trial was performed using an opaque Perspex Y-maze and the angle between each wall is 120°. Each mouse was placed from the top of arm A and explored freely in the maze for 3 min. After each exploration, the Y-maze was cleaned with 70% ethanol. Spontaneous alternation was quantified using the video tracking system and was defined as a successive entry into three different arms. The alternation ratio is the main index to evaluate spatial working memory which is calculated as correct alternation frequency/total alternation frequency×100%.

#### Morris water maze (MWM)

The MWM was conducted with a circular pool with a radius of 60 cm, a height of 50 cm and a depth of 25 cm. The pool water was made opaque by water-soluble non-toxic white paint and maintained at a temperature of 22 ± 1 °C. The whole experiment was divided into a five-day learning task and a one-day probe trial test. During the learning task, each mouse was subjected to four training trials to find a hidden platform 1.5 cm below the water surface within 60 s for 5 days consecutively. Each mouse was allowed to adapt to the platform for 10 s if it could find the platform. If the mouse failed to find the platform within 60 s, it would be guided to the platform and stay there for 10 s. Twenty-four hours after the last learning task, the platform was removed and a probe trial test was conducted. Mice were allowed to swim for 60 s and their performance was recorded to evaluate memory retention using the automated tracking system.

### Statistical analysis

All data were presented by at least three biologically independent experiments. Statistical analysis was performed using GraphPad Prism 8.0 and all data were shown as mean ± standard error of mean (SEM). Two-sided unpaired Student’s *t*-test and one-way ANOVA followed by post-hoc Tukey test were used to compare the difference between two independent samples. The comparisons among groups were analyzed by two-way ANOVA with post-hoc Tukey adjustment. The correlations were calculated by Pearson correlation analysis. The statistical analyses used for the different experiments were described in the respective figure legends and all image acquisition and quantification were conducted in a blinded manner. Reasonable sample sizes were determined according to our previous studies and other similar publications for different experiments. All statistical analyses were deemed significant when *P* < 0.05 and the *P*-value was reported on the graph highlighted comparison. 

## Electronic supplementary material

Below is the link to the electronic supplementary material.

Supplementary Material 1

Supplementary Material 2

Supplementary Material 3

Supplementary Material 4

Supplementary Material 5

Supplementary Material 6

Supplementary Material 7

Supplementary Material 8

Supplementary Material 9

Supplementary Material 10

Supplementary Material 11

Supplementary Material 12

Supplementary Material 13

## Data Availability

All data generated or analyzed during this study are included in this published article and its supplementary information. The datasets used and analyzed during the current study available from the corresponding author on reasonable request.

## Abbreviations

AAV: Adeno-associated virus
Aβ: Amyloid beta
AD: Alzheimer’s disease
Ang II: Angiotensin II
AQP4: Aquaporin 4
BBB: Blood-brain barrier
bEnd.3: Brain-derived endothelial cells.3
BMECs: Brain microvascular cells
CEEVs: Cerebrovascular endothelial extracellular vesicles
CNS: Central nervous system
CSF: Cerebral spinal fluid
DEGs: Differential genes
DG: Dentate gyrus
eGFP: Enhanced green fluorescent protein
ENG: Endoglin
EVs: Extracellular vesicles
EVG: Elastica van Gieson
GFAP: Glial fibrillary acidic protein
HCMEC/D3: Human cortical microvessels endothelial cells/D3
Iba1: Ionized calcium-binding adaptor molecule 1
IL-3: Interleukin 3
IL-6: Interleukin 6
LPS: Lipopolysaccharide
MCI: Mild cognitive impairment
MoCA: Montreal Cognitive Assessment
MWM: Morris water maze
NC: Non-demented control
NOR: Novel object recognition
NVU: Neurovascular unit
S100B: S100 calcium binding protein B
TGFβ: Transforming growth factor beta
TGFBR: TGFβ receptor
TNF-α: Tumor necrosis factor-alpha
VEGF: Vascular endothelial growth factor
VIM: Vimentin
WT: Wild type
ZO-1: Zonula occludens-1
