# Supplementary material for: Endothelium-specific endoglin triggers astrocyte reactivity via extracellular vesicles in a mouse model of Alzheimer’s disease
Source: Mol Neurodegener. 2025 Jul 23;20:84. doi: 10.1186/s13024-025-00875-4 (PMC12285072; doi:10.1186/s13024-025-00875-4)
Supplement: Supplementary file 1 — Supplementary Material 1 [file 13024_2025_875_MOESM1_ESM.doc]

**Supplementary Methods and Materials**

**Cell cultures transfection and reagents**

HCMEC/D3 cell line was purchased from Cell Resource Center, Shanghai Institute of Biological Sciences, Chinese Academy of Sciences (GNHu68, China), with human cell STR identification. bEnd.3 cell line was purchased from FuHeng Biology (#FH0356, China) with mouse cell STR identification. Cells were cultured in Dulbecco’s modified Eagle’s medium (DMEM, Thermo fisher scientific, #11965092, USA) supplemented with 10% heat-inactivated FBS (Thermo fisher scientific, #10099141C, USA) and 1% penicillin-streptomycin mix (Thermo Fisher Scientific, #15140122, USA). Cell experiments were carried out within 5 to 30 passages. HCMEC/D3 and bEnd.3 cells were digested with 0.25% EDTA Trypsin (Thermo fisher scientific, #25200072, USA) for 2 min and then centrifuged at 1,000 rpm for 5 min. The cells are counted and then seeded in the plates. All cells were cultured at 37°C in a humidified 5% CO_2_ atmosphere. Transfection was performed when the HCMEC/D3 cell confluence reached 80%. The ENG-overexpression plasmid and truncated plasmid were purchased from Sinobiological (#HG10149-CH, China). The shRNA targeting ENG (shENG) utilized *in vitro* was consistent with the AAV-shENG applied *in vivo* which was listed in Supplementary Table 3. The ENG-overexpression plasmid was transfected into cells by using Lipofectamine 3000 (Thermo fisher scientific, #L3000015, USA) according to the manufacturer’s instructions.

**Two-photon imaging *in vivo***

Surgical procedures closely followed established protocols. Briefly, mice were anesthetized with isoflurane (4-5% for induction, 1-1.5% for maintenance), head-fixed with blunt ear bars and kept at 36-37 °C on a custom surgical bed (Thorlabs, USA). Mice were implanted with cranial windows. After recovery from cranial window surgery, MCAAD-3 (Abcam, #ab216983, USA) (dissolved in 80% propylene glycol and 20 % DMSO, 60 μg/mL, 50 μL) was tail-vein injected to label Aβ, and TRITC-Dextran dye (Xarxbio, #R-FD-039, China) (dissolved in normal saline, 100 mg/mL, 150 μL) was tail-vein injected to label blood vessels. Two-photon imaging was performed after 30 min and captured 15 μm, 2 μm z-resolution, 1024×1024 pixel stacks every minute for 10-20 min. The images were analyzed by ImageJ to observe and record cerebral vascular morphology and cerebral blood flow velocity. The Skeleton analysis plug-in was then used to measure blood vessel diameter and the number of blood vessel branches. Matlab software was used to process the maximum projected signal of blood vessels, calculate the blood vessel diameter, and obtain the distribution histogram of blood vessel diameter. The Aβ plaques were processed by Imaris software, and the signal of Aβ plaques was rendered 3D by the Surface function. The total volume of Aβ plaques was calculated, and the number of Aβ plaques in different volumes was counted.

**ELISA**

Mouse tissues or cell pellets were lysed and homogenized in RIPA buffer with protease inhibitors (APExBIO, #K1007, USA), and ELISA assay was carried out according to the instructions and the plates were read at 450 nm on a Synergy MX Plate Reader (BioTek). The ELISA kits used in this study were shown as follows: Human endoglin ELISA kit (Thermo Fisher Scientific, #EHENG, USA), mouse endoglin ELISA kit (USCNK, #SEA980Mu, China). S100B ELISA kit (USCNK, #SEA567Mu, China), IL-6 ELISA kit (MultiSciences, #70-EK206/3, China), IL-3 ELISA kit (MultiSciences, #70-EK268/2, China) and VEGF ELISA kit (MultiSciences, #70-EK283/2, China). Hippocampus tissue of mice was homogenized in PBS to get the soluble protein and in guanidine to extract the insoluble component. Aβ_40_ and Aβ_42_ levels were determined according to the manufacturer's instructions with Aβ_40_ ELISA kit (CUSABIO, #CSB-E10684h, China) and Aβ_42_ ELISA kit (CUSABIO, #CSB-E08299h, China) respectively.

**Nanoparticle Tracking Analysis (NTA)**

Concentration and particle size of purified extracellular vesicles (EVs) were measured by Nanosight (Malvern system Ltd., #NS300, U.K.). Briefly, each sample, once properly diluted, was recorded for 60 s with a detection threshold set at maximum. Temperature was monitored throughout the measurements. Vesicle size distribution and an estimated concentration of NTA profiles were obtained from the given raw data files.

**Transmission electron microscopy**

The EVs samples, retrieved from a -80℃ freezer, were initially placed in an ice box to thaw and then briefly centrifuged. Using a pipette, 15 μL of the EVs sample was carefully placed onto a copper grid and allowed to stand for 1 min, ensuring the grid wasn't damaged during handling. A filter paper was then used to remove excess liquid from the grid, followed by the application of 15 μL of 2% uranyl acetate stain at room temperature for 1 min. After staining, the excess stain was removed with filter paper, and the stained samples were baked under a lamp for 10 min before being observed, photographed, and the images preserved.

**Golgi staining**

Referenced to our established methods ([*Li JB, et al*. 2023](https://translationalneurodegeneration.biomedcentral.com/articles/10.1186/s40035-022-00334-w)), the Golgi staining technique and dendritic spine analysis were following steps: Initially, freshly prepared brain tissue was impregnated with a potassium dichromate solution, allowing the neurons to become chemically fixed. This was followed by immersion in a silver nitrate solution, which precipitated silver chromate within the neurons, rendering them visible. After adequate impregnation, the tissue was meticulously sectioned using a microtome. The sections were then carefully mounted onto slides and dehydrated through a graded series of ethanol to xylene. Finally, the sections were cover-slipped with a suitable mounting medium, resulting in a detailed visualization of neuronal morphology for microscopic examination. Finally, the sections were stained for histological analysis and observed with Leica Qwin software (Leica, Germany). ImageJ software was used to analyze the number of spines and the total dendritic length. Distal dendrites > 50 µm from the soma were selected for the measurement of spine density.

**CCK-8 assay**

Cells were plated at a density of 2×10^3^ cells per well on 96-well plates in 100 μL of culture medium. At the indicated time, CCK-8 (10 µL, Beyotime, #C0042, China) was added to each well and incubated for an additional 24 h. The OD was measured with a Multiscan spectrum (Thermo Fisher Scientific, VRIOSKAN FLASH, USA) at the wave length of 450 nm.

**Supplementary Figure Legends**

**Supplementary Figure 1. Ang II induces vascular injury and cognitive dysfunction in mice.**

**a, b** Representative images (**a**) and the quantification of capillary area ratio, number of vascular incisions and elastic fiber thickness (**b**) by Elastica van Gieson (EVG) staining in the hippocampal dentate gyrus of WT-Sham and WT-Ang II mice (n = 5 mice per group). **c, d** The representative heat maps (**c**) and the moving speed in NOR, Y maze and MWM tests (**d**) of WT-Sham (n = 7 mice per group) and WT-Ang II mice (n = 6 mice per group). **e** The expression of AT1R and AT2R in brain cells. Data supported by [The Human Protein Atlas](https://www.proteinatlas.org/). **f** Cell viability of astrocytes treated by Ang II, 24 h (n = 6 biologically independent experiments). **g** mRNA levels of *GFAP* in astrocytes treated by Ang II, 24 h (n = 6 biologically independent experiments). **h** Immunoblotting and the quantification of GFAP in astrocytes treated by Ang II, 24 h (n = 3 biologically independent experiments). **i, j** IL-6, VEGF (**i**) and S100B (**j**) levels in the serum of WT-Sham and WT-Ang II mice (n = 3 mice per group). **k** Representative fluorescence images of AQP4 and Lectin colocalization in the hippocampal dentate gyrus of WT-Sham and WT-Ang II mice (n = 3 mice per group). Data are shown as means ± SEM, ns: non-significant, and the *P*-value was reported on the graph highlighted comparison by unpaired two-sided *t*-test (**b-d, h-k**) or one-way ANOVA with post-hoc Tukey adjustment (**f**). AT1R: Angiotensin type I receptor, AT2R: Angiotensin type II receptor, Ang II: Angiotensin II, NOR: new object recognition test, MWM: Morris water maze test.

**Supplementary Figure 2. Injured** **BMECs-derived EVs promote astrocyte reactivity.**

**a** Schematic of cerebrovascular endothelial extracellular vesicles (CEEVs) extraction and purification. **b, c** Electron microscopy (**b**) and nanoparticle tracking analysis (NTA) (**c**) confirmed CEEVs. **d** Primary astrocytes could receive CEEVs from HCMEC/D3 cells. **e** Immunoblotting and the quantification of GFAP in astrocytes treated by injured HCMEC/D3-CEEVs (n = 3 biologically independent experiments). **f** Immunoblotting and the quantification of GFAP in astrocytes treated by CEEVs with or without Triton and papain (n = 4 biologically independent experiments). Data are shown as means ± SEM, and the *P*-value was reported on the graph highlighted comparison by unpaired two-sided *t*-test (**e**) or one-way ANOVA with post-hoc Tukey adjustment (**f**).

**Supplementary Figure 3. Endothelial cell-specific Endoglin (ENG) is a potential communicator from the vasculature to astrocytes.**

**a** Venn analysis of HCMEC-DEGs and specific genes of different neutral cells (FPKM fold change > 4). **b** Heatmap of FPKM in expression of 35 candidate genes extracted from first round screening ([*Zhang et al*., 2014](https://pmc.ncbi.nlm.nih.gov/articles/PMC4152602/)), which are preferentially expressed in endothelial cells over other cell types. ECs: endothelial cells. **c** t-SNE maps of the brain transcriptomic map constructed from the data set reported in [*Tabula Muris Consortium, et al*., 2018](https://pmc.ncbi.nlm.nih.gov/articles/PMC6642641/). Cells for brain visualized with tSNE, colored by cell type. Cell types were determined by differential gene expression of known markers between clusters (up). ENG was mainly expressed in endothelial cell cluster (down). **d** mRNA levels of *ENG* in HCMEC/D3 and astrocytes (n = 6 biologically independent experiments). **e** Immunoblotting and the quantification of ENG in HCMEC/D3 and primary mouse astrocytes (n = 6 biologically independent experiments). **f** ENG expression in the hippocampus of WT and APP/PS1 mice (n = 5 mice per group). **g** The ENG levels in the serum of different sexes of AD patients (n = 18) and Non-demented controls (NC) (n = 15). **h, i** The age (**h**) and MoCA score (**i**) of AD patients and NC. Data are shown as means ± SEM, ns: non-significant, and the *P*-value was reported on the graph highlighted comparison by unpaired two-sided *t*-test. MoCA: Montreal Cognitive Assessment.

**Supplementary Figure 4. ENG is expressed in vascular endothelium and highly conserved.**

**a** Conservative analysis of ENG amino acid sequence by [PSI-Coffee](https://tcoffee.crg.eu/apps/tcoffee/result?rid=91357a9d) and transmembrane structure between human and mouse by [PSI/TM-Coffee](https://tcoffee.crg.eu/apps/tcoffee/result?rid=5640a663) alignment.

**Supplementary Figure 5. Vascular injury enhances ENG expression and release in BMECs.**

**a** Schematic of the HCMEC/D3 treatment. **b** mRNA levels of *EN*G in HCMEC/D3 treated with Ang II (n = 6 biologically independent experiments). **c** Immunoblotting and the quantification of ENG protein levels in HCMEC/D3 treated with Ang II (n = 3 biologically independent experiments), while GAPDH as loading controls. **d, e** mRNA levels of *ENG* in HCMEC/D3 cells (**d**) and ENG protein levels in HCMEC/D3 medium treated by LPS, IL-6 or TNF-α for 24 h (n = 6 biologically independent experiments) (**e**). **f** ENG protein levels in the serum of mice aged 2, 9, and 16 months (n = 3 mice per group). **g** ENG protein levels in the serum of WT-Sham and WT-Ang II mice (n = 6 mice per group). **h** Astrocytes received ENG-contained CEEVs. **i** Schematic of astrocytes and HCMEC/D3 co-culture system (AST & HC Co-culture). Data are shown as means ± SEM, ns: non-significant, and the *P*-value was reported on the graph highlighted comparison by unpaired two-sided *t*-test (**b, c, g**) or one-way ANOVA with post-hoc Tukey adjustment (**d-f**). AST: astrocyte, HC: HCMEC/D3.

**Supplementary Figure 6. ENG regulates astrocytes via TGFBRI signaling pathway.**

**a** Schematic of TGFβ/ENG/TGFBR/Smad3 signal pathway. **b** Representative images and co-localization analysis of ENG on astrocyte cytomembrane of AST & HC Co-culture. **c** TGFBRI and TGFBRII levels in astrocytes in single-cell database supported by [The Human Protein Atlas](https://www.proteinatlas.org/). **d** Immunoblotting of TGFBRI and TGFBRII levels in astrocytes (n = 6). **e** Immunoprecipitation and immunoblot analysis of ENG and TGFBRII in the astrocytes of AST & HC Co-culture. **f** The key marker genes of reactive astrocytes are in ENG/TGFBR/Smads signaling pathway. Data supported by [STRING](https://cn.string-db.org/) database. TGFBR: TGFβ receptor.

**Supplementary Figure 7. AAV-shENG specifically decreased ENG expression in BMECs.**

**a** Co-localization analysis of eGFP and Lectin in the hippocampus 31 days after AAV-shENG injection. **b** Immunoblotting and the quantification of ENG protein levels in the hippocampus of WT, APP/PS1 and APP/PS1-shENG mice (n = 5 mice per group). **c** mRNA levels of *ENG* in the hippocampus of WT, APP/PS1 and APP/PS1-shENG mice (n = 5 mice per group). **d** Immunoblotting and the quantification of AQP4 and Lectin colocalization in the hippocampal dentate gyrus of APP/PS1-shCon and APP/PS1-shENG mice (n = 3 mice per group). Data are shown as means ± SEM, and the *P*-value was reported on the graph highlighted comparison by unpaired one-way ANOVA with post-hoc Tukey adjustment (**b, c**) and unpaired two-sided *t*-test (**d**).

**Supplementary Figure 8. ENG deficiency in endothelium inhibits inflammatory response.**

**a-d** Representative images (**a**), quantification of ENG spots on per astrocyte (**b**), quantification of GFAP level (**c**) and correlation analysis between GFAP and ENG spots on per astrocyte from 3 mice per group (**d**) in the hippocampal dentate gyrus of APP/PS1-shCon and APP/PS1-shENG mice. **e, f** Representative images (**e**) and quantification of Iba1 (**f**) in the hippocampal dentate gyrus of APP/PS1-shCon and APP/PS1-shENG mice (n = 3 mice per group). **g** mRNA levels of *ENG* in the hippocampus of APP/PS1-shCon and APP/PS1-shENG mice (n = 6 mice per group). **h, i** Heatmap of DEGs (**h**) and GO analysis of DEGs (**i**) in the hippocampus of APP/PS1-shCon and APP/PS1-shENG mice. Data are shown as means ± SEM, and the *P*-value was reported on the graph highlighted comparison by unpaired two-sided *t*-test.

**Supplementary Figure 9. ENG deficiency improves cognitive dysfunction and AD pathology.**

**a** The body weight of APP/PS1-Sham (n = 8 mice per group), APP/PS1-shCon (n = 8 mice per group) and AAV-shENG mice (n = 9 mice per group). **b, c** Motion trail (**b**) and the new object recognition index (**c**) of NOR. **d** The alternation and the total entry number of Y maze. **e** The time in target quadrant and the swimming velocity in MWM. **f** Two-photon analysis of cerebral vascular density and morphology in APP/PS1-shCon and APP/PS1-shENG mice (n = 3 mice per group). **g** Representative images and quantification of ZO-1 and Lectin in the hippocampus of APP/PS1-shCon and APP/PS1-shENG mice (n = 3 mice per group). Data are shown as means ± SEM, ns: non-significant, and the *P*-value was reported on the graph highlighted comparison by two-way ANOVA with post-hoc Tukey adjustment (**a**) or one-way ANOVA with post-hoc Tukey adjustment (**c-e**) and unpaired two-sided *t*-test (**f, g**).

**Supplementary Figure 10. Carotuximab improves cognitive dysfunction in APP/PS1 mice.**

**a** The NOR index and moving speed in NOR test. **b** The total entry number of spontaneous alternations in Y maze. **c** The swimming velocity during the probe trial test in MWM test. **d** The body weight of WT-Sham (n = 9 mice per group), WT-Carotuximab (n = 8 mice per group), APP/PS1-Sham (n = 9 mice per group) and APP/PS1-Carotuximab (n = 9 mice per group) mice. **e** The protein level of soluble and insoluble Aβ_40_ in the hippocampus of APP/PS1-Sham and APP/PS1-Carotuximab mice (n = 9 mice per group). Data are shown as means ± SEM, ns: non-significant, and the *P*-value was reported on the graph highlighted comparison by two-way ANOVA with post-hoc Tukey adjustment (**a-d**) or unpaired two-sided *t*-test (**e**).
