# Supplementary figures and images for: Endothelium-specific endoglin triggers astrocyte reactivity via extracellular vesicles in a mouse model of Alzheimer’s disease
Source: Mol Neurodegener. 2025 Jul 23;20:84. doi: 10.1186/s13024-025-00875-4 (PMC12285072; doi:10.1186/s13024-025-00875-4)

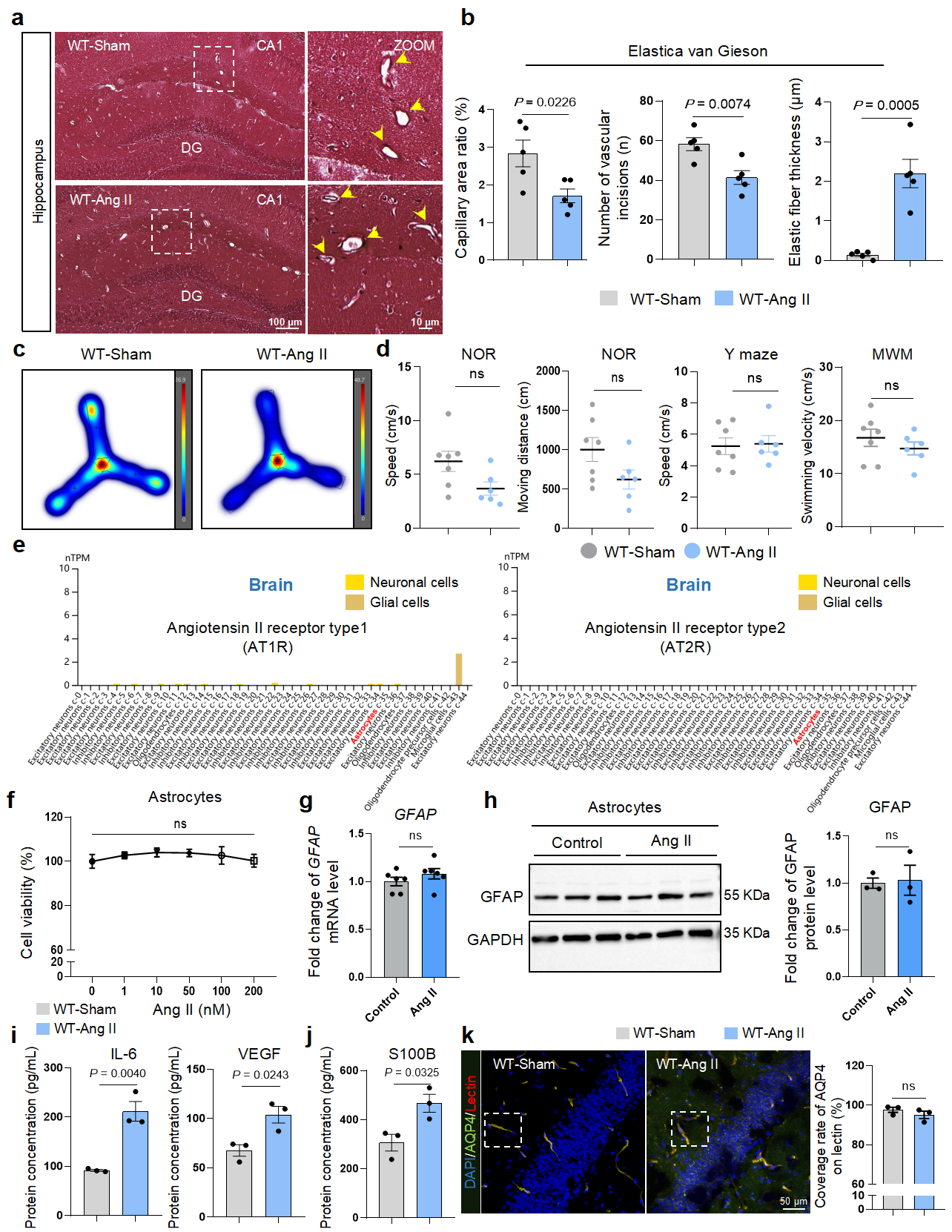

Supplement: Supplementary file 2 — Supplementary Material 2 [file 13024_2025_875_MOESM2_ESM.tif]

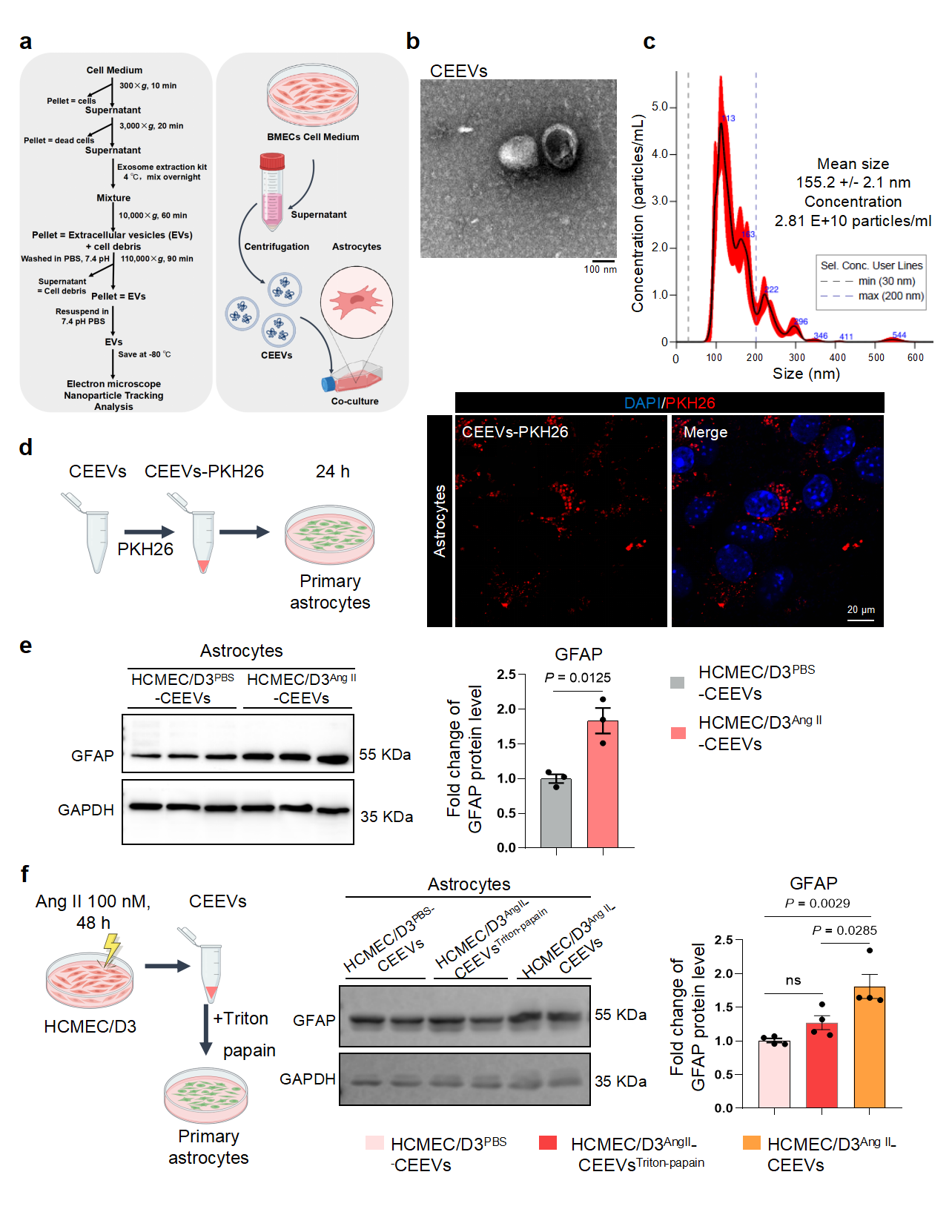

Supplement: Supplementary file 3 — Supplementary Material 3 [file 13024_2025_875_MOESM3_ESM.tif]

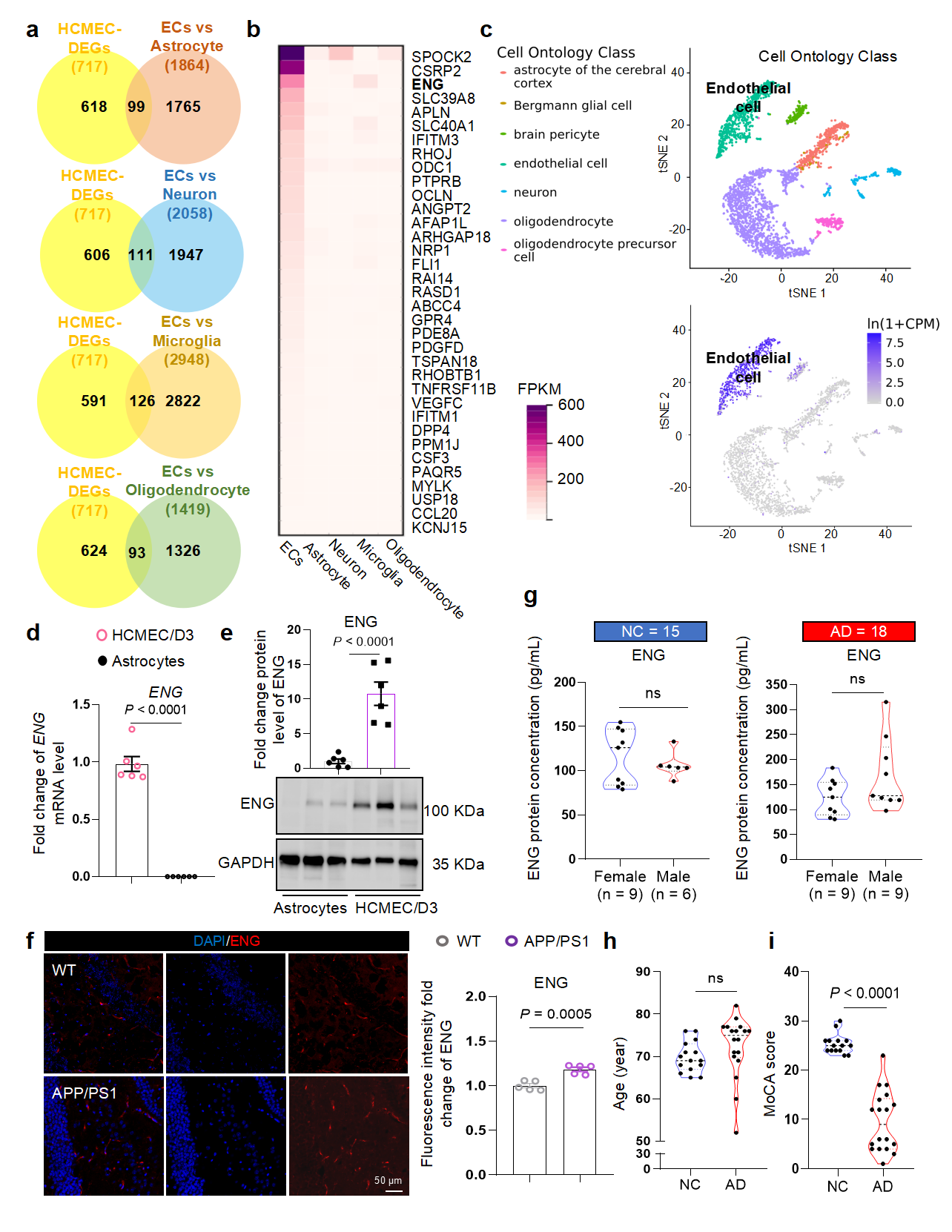

Supplement: Supplementary file 4 — Supplementary Material 4 [file 13024_2025_875_MOESM4_ESM.tif]

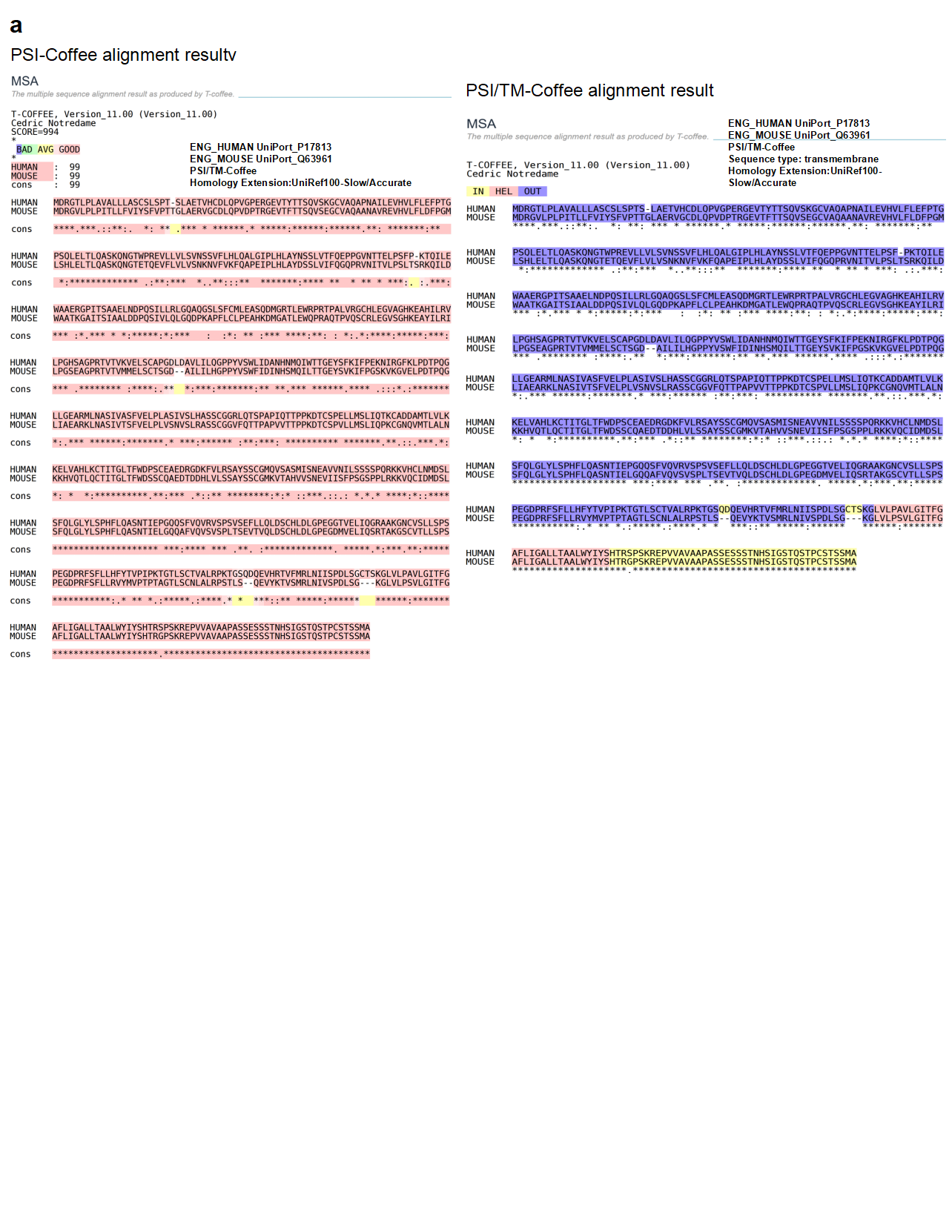

Supplement: Supplementary file 5 — Supplementary Material 5 [file 13024_2025_875_MOESM5_ESM.tif]

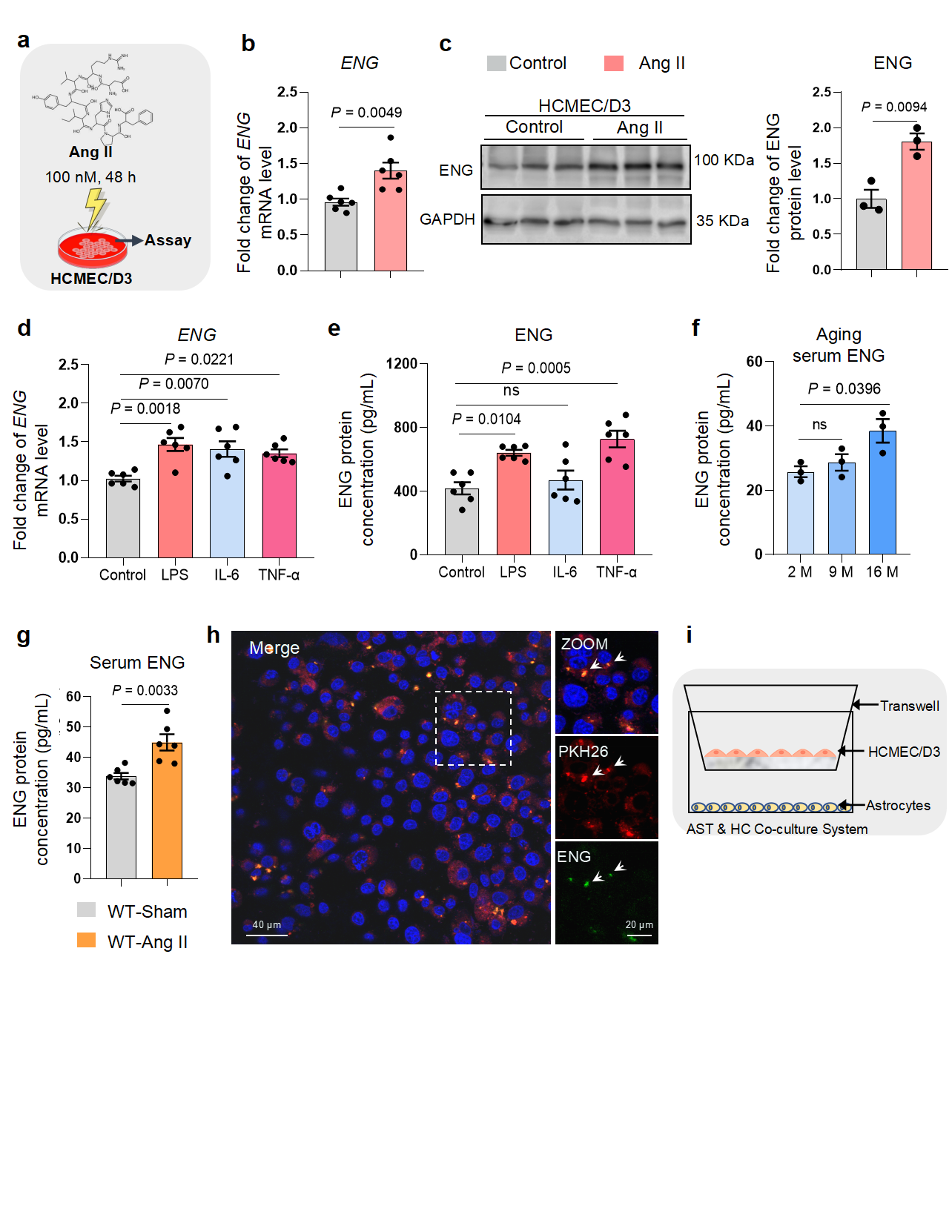

Supplement: Supplementary file 6 — Supplementary Material 6 [file 13024_2025_875_MOESM6_ESM.tif]

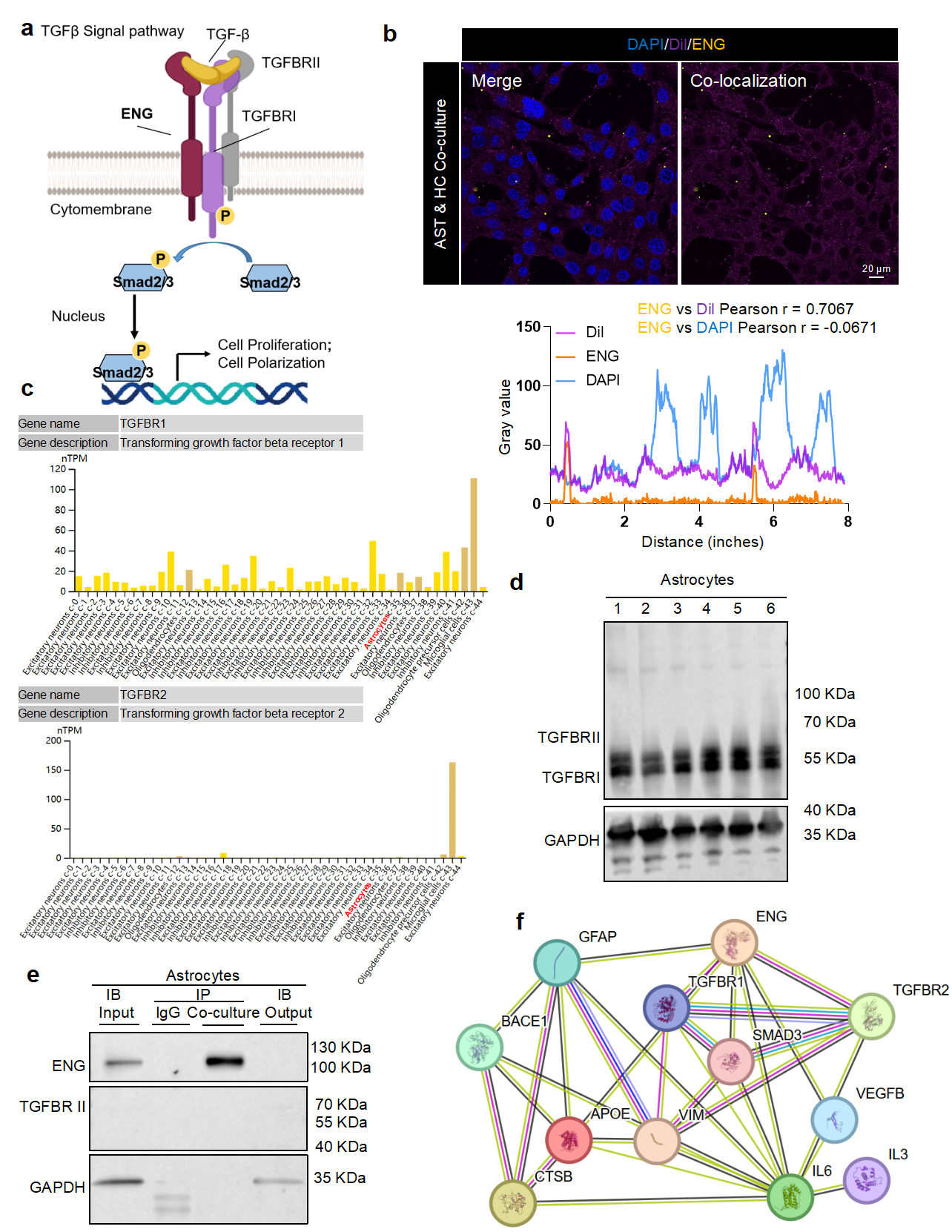

Supplement: Supplementary file 7 — Supplementary Material 7 [file 13024_2025_875_MOESM7_ESM.tif]

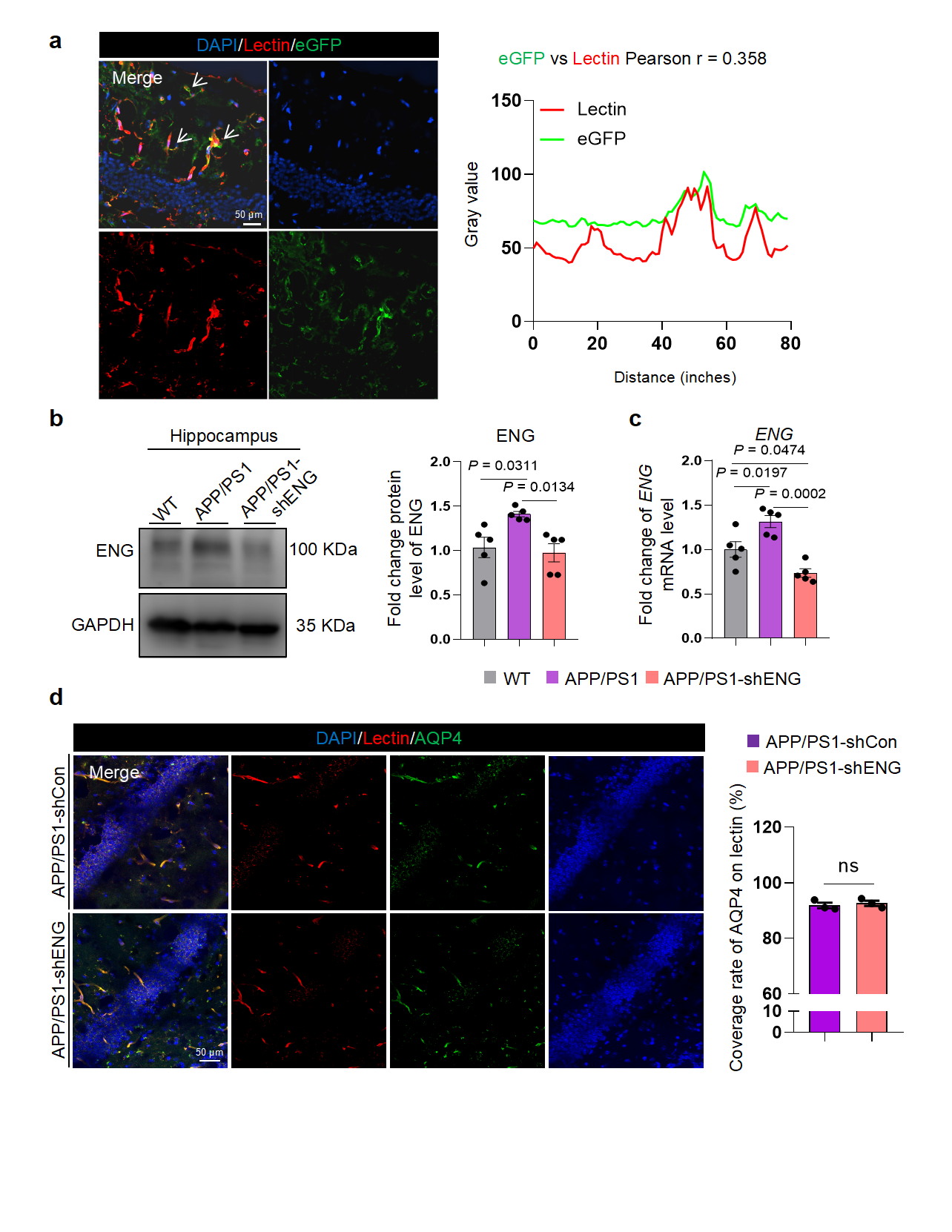

Supplement: Supplementary file 8 — Supplementary Material 8 [file 13024_2025_875_MOESM8_ESM.tif]

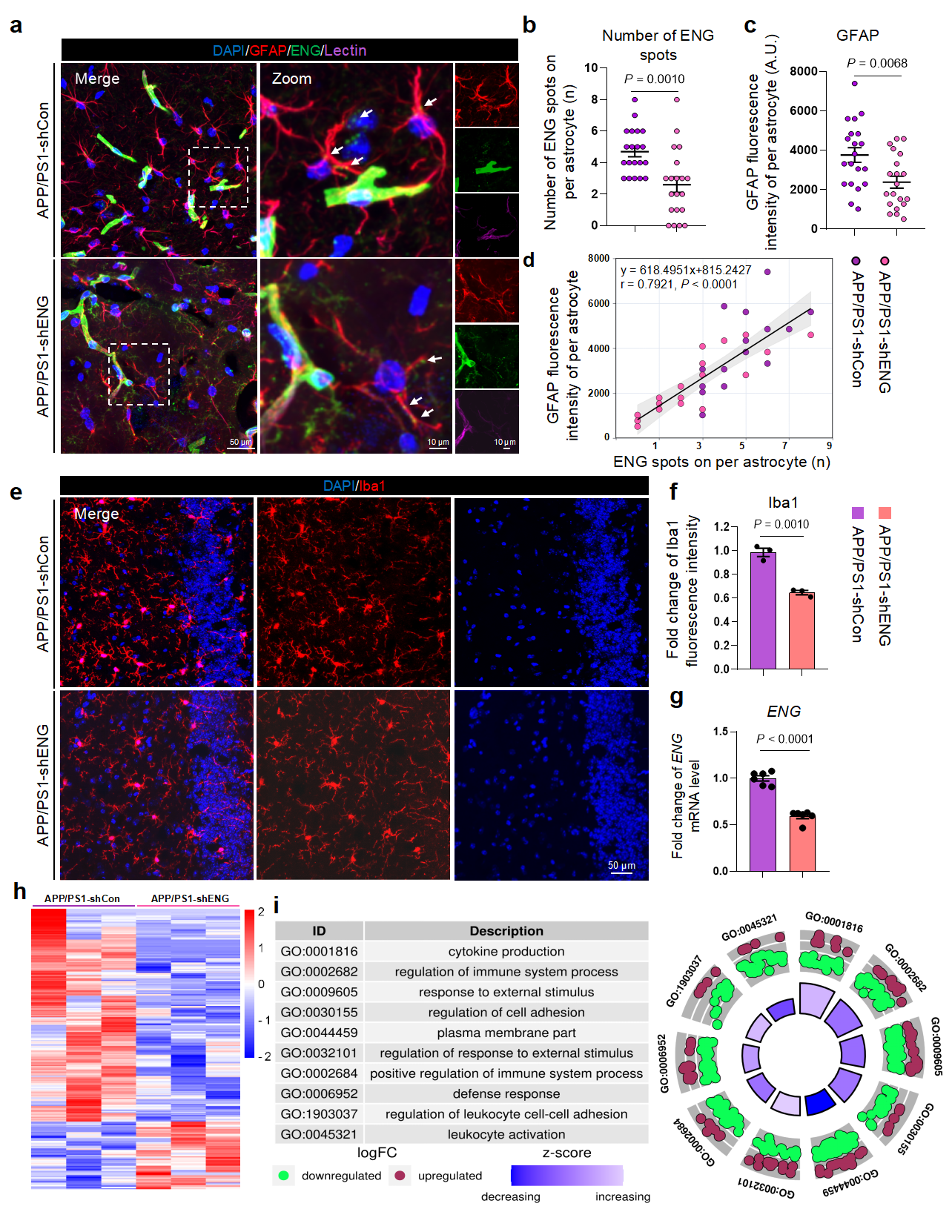

Supplement: Supplementary file 9 — Supplementary Material 9 [file 13024_2025_875_MOESM9_ESM.tif]

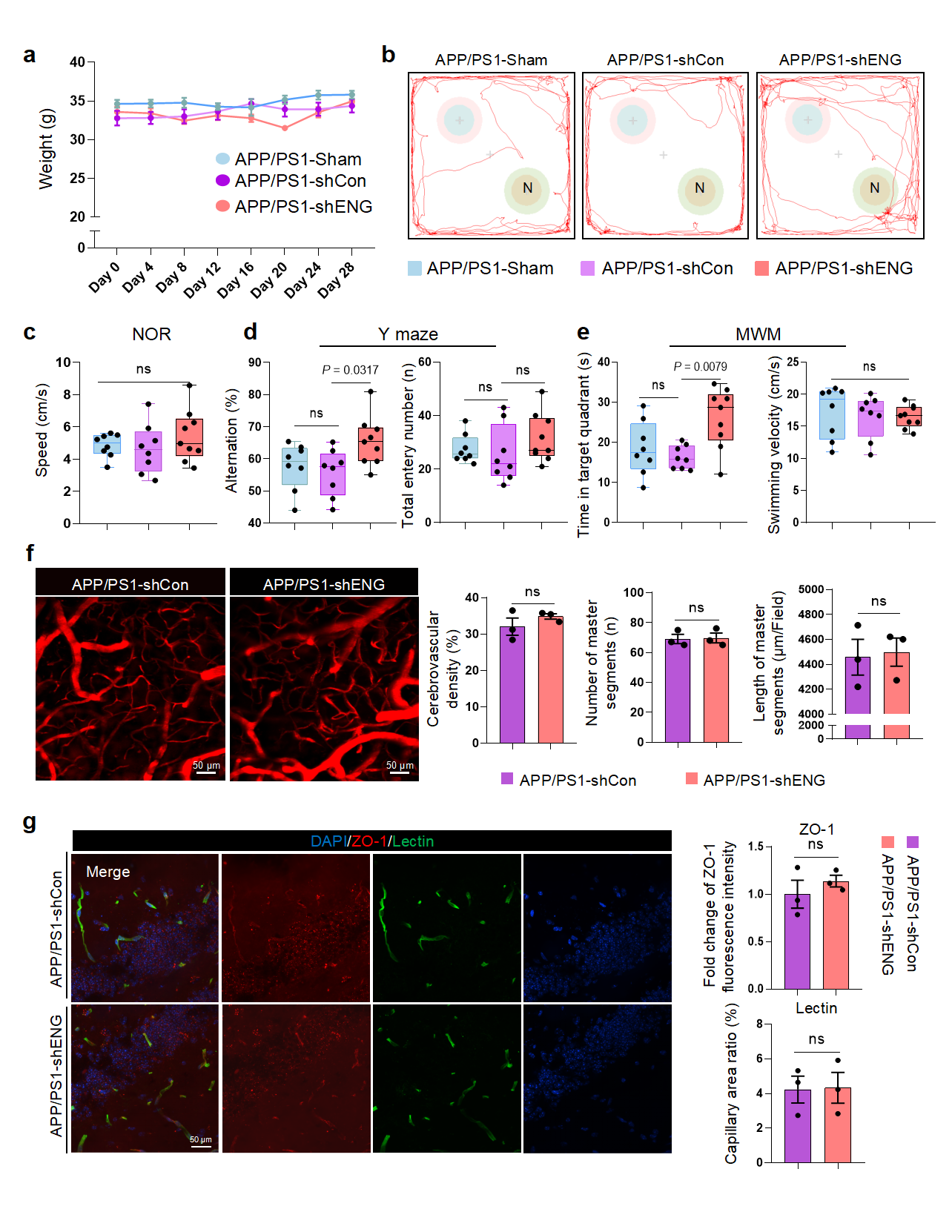

Supplement: Supplementary file 10 — Supplementary Material 10 [file 13024_2025_875_MOESM10_ESM.tif]

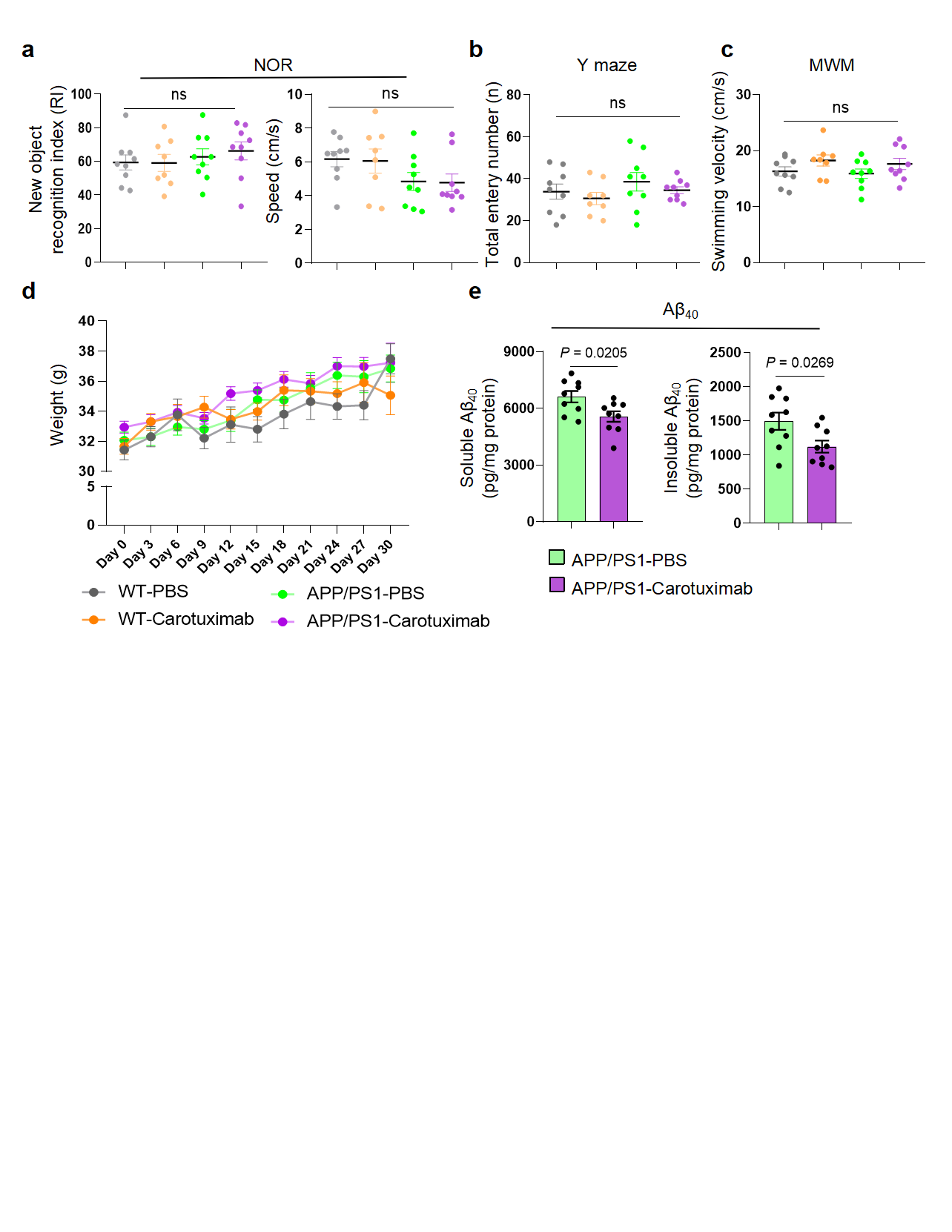

Supplement: Supplementary file 11 — Supplementary Material 11 [file 13024_2025_875_MOESM11_ESM.tif]
