# Supplementary material for: Endothelium-specific endoglin triggers astrocyte reactivity via extracellular vesicles in a mouse model of Alzheimer’s disease
Source: Mol Neurodegener. 2025 Jul 23;20:84. doi: 10.1186/s13024-025-00875-4 (PMC12285072; doi:10.1186/s13024-025-00875-4)
Supplement: Supplementary file 12 — Supplementary Material 12 [file 13024_2025_875_MOESM12_ESM.doc]

**Supplementary Table 1.** **Differential genes enriched in Ang II-treated vascular endothelial cells**

| **No.** | **Gene Symbol** | **Description** |
| --- | --- | --- |
| 1 | *ABCC4* | ATP Binding Cassette Subfamily C Member 4 |
| 2 | *AFAP1L1* | Actin Filament Associated Protein 1 Like 1 |
| 3 | *ANGPT2* | Angiopoietin-2 |
| 4 | *APLN* | Apelin |
| 5 | *ARHGAP18* | Rho GTPase Activating Protein 18 |
| 6 | *CCL20* | C-C Motif Chemokine Ligand 20 |
| 7 | *CSF3* | Colony Stimulating Factor 3 |
| 8 | *CSRP2* | Cysteine And Glycine Rich Protein 2 |
| 9 | *DPP4* | Dipeptidyl Peptidase-4 |
| 10 | *ENG* | Endoglin |
| 11 | *FLI1* | Fli-1 Proto-Oncogene, ETS Transcription Factor |
| 12 | *GPR4* | G Protein-Coupled Receptor 4 |
| 13 | *IFITM1* | Interferon Induced Transmembrane Protein 1 |
| 14 | *IFITM3* | Interferon Induced Transmembrane Protein 3 |
| 15 | *KCNJ15* | Potassium Inwardly Rectifying Channel Subfamily J Member 15 |
| 16 | *MYLK* | Myosin Light Chain Kinase |
| 17 | *NRP1* | Neuropilin 1 |
| 18 | *OCLN* | Occludin |
| 19 | *ODC1* | Ornithine Decarboxylase 1 |
| 20 | *PAQR5* | Progestin And AdipoQ Receptor Family Member 5 |
| 21 | *PDE8A* | Phosphodiesterase 8A |
| 22 | *PDGFD* | Platelet Derived Growth Factor D |
| 23 | *PPM1J* | Protein Phosphatase, Mg2+/Mn2+ Dependent 1J |
| 24 | *PTPRB* | Protein Tyrosine Phosphatase Receptor Type B |
| 25 | *RAI14* | Retinoic Acid Induced 14 |
| 26 | *RASD1* | Ras Related Dexamethasone Induced 1 |
| 27 | *RHOBTB1* | Rho Related BTB Domain Containing 1 |
| 28 | *RHOJ* | Ras Homolog Family Member J |
| 29 | *SLC39A8* | Solute Carrier Family 39 Member 8 |
| 30 | *SLC40A1* | Solute Carrier Family 40 Member 1 |
| 31 | *SPOCK2* | SPARC (Osteonectin), Cwcv And Kazal-like Domains Proteoglycan 2 |
| 32 | *TNFRSF11B* | TNF Receptor Superfamily Member 11b |
| 33 | *TSPAN18* | Tetraspanin 18 |
| 34 | *USP18* | Ubiquitin Specific Peptidase 18 |
| 35 | *VEGFC* | Vascular Endothelial Growth Factor C |

**Supplementary** Table 2. Patient information sheet

| **Group** | **Source** | **Number** | **Age** | **Gender** | **MoCA** | **ENG (pg/mL)** | **Other neurological diseases** |
| --- | --- | --- | --- | --- | --- | --- | --- |
| NC | NCT05667935 | 1 | 65 | Female | 30 | 154.87 | None |
|  | NCT05667935 | 2 | 66 | Female | 25 | 89.98 | None |
|  | NCT05667935 | 3 | 67 | Female | 26 | 85.60 | None |
|  | NCT05667935 | 4 | 69 | Female | 29 | 82.04 | None |
|  | NCT05667935 | 5 | 70 | Female | 24 | 145.50 | None |
|  | NCT05667935 | 6 | 71 | Female | 26 | 148.59 | None |
|  | NCT05667935 | 7 | 73 | Female | 26 | 126.04 | None |
|  | NCT05667935 | 8 | 74 | Female | 23 | 79.11 | None |
|  | NCT05667935 | 9 | 76 | Female | 24 | 131.95 | None |
|  | NCT05667935 | 10 | 65 | Male | 25 | 103.30 | None |
|  | NCT05667935 | 11 | 68 | Male | 25 | 88.20 | None |
|  | NCT05667935 | 12 | 68 | Male | 24 | 103.45 | None |
|  | NCT05667935 | 13 | 69 | Male | 26 | 104.95 | None |
|  | NCT05667935 | 14 | 71 | Male | 24 | 132.97 | None |
|  | NCT05667935 | 15 | 76 | Male | 23 | 105.84 | None |
| AD | NCT05667935 | 1 | 60 | Female | 3 | 183.43 | None |
|  | NCT05667935 | 2 | 74 | Female | 5 | 157.77 | None |
|  | NCT05667935 | 3 | 79 | Female | 6 | 100.78 | None |
|  | NCT05667935 | 4 | 65 | Female | 13 | 124.75 | None |
|  | NCT05667935 | 5 | 76 | Female | 12 | 151.69 | None |
|  | NCT05667935 | 6 | 77 | Female | 12 | 141.63 | None |
|  | NCT05667935 | 7 | 71 | Female | 14 | 83.28 | None |
|  | NCT05667935 | 8 | 74 | Female | 17 | 95.53 | None |
|  | NCT05667935 | 9 | 69 | Female | 17 | 80.47 | None |
|  | NCT05667935 | 10 | 71 | Male | 1 | 315.51 | None |
|  | NCT05667935 | 11 | 77 | Male | 4 | 246.77 | None |
|  | NCT05667935 | 12 | 77 | Male | 4 | 203.26 | None |
|  | NCT05667935 | 13 | 76 | Male | 4 | 118.98 | None |
|  | NCT05667935 | 14 | 76 | Male | 5 | 127.71 | None |
|  | NCT05667935 | 15 | 82 | Male | 6 | 97.55 | None |
|  | NCT05667935 | 16 | 70 | Male | 12 | 123.55 | None |
|  | NCT05667935 | 17 | 76 | Male | 15 | 119.24 | None |
|  | NCT05667935 | 18 | 52 | Male | 23 | 171.51 | None |

*NC: Non-demented control, AD: Alzheimer’s disease, ENG: Endoglin, MoCA: Montreal Cognitive Assessment.

**Supplementary Table 3. Sequence of adeno-associated virus**

|  | **Description** | **Sequence 5’ to 3’** |
| --- | --- | --- |
| **AAV-shCon** | Target sequence | TTCTCCGAACGTGTCACGT |
|  | shRNA oligo sequence | TCGATTCTCCGAACGTGTCACGT |
|  | AAV sequence | TTTCCACGTTCTCTCGGCATGGACGAGCTGTACAAGTGAGCGAATTGAATGAGGCTTCAGTACTTTACAGAATCGTTGCCTGCACATCTTGGAAACACTTGCTGGGATTACTTCTTCAGGTTAACCCAACAGAAGGCTCGAGAAGGTATATTGCTGTTGACAGTGATTTCGATTCTCCGAACGTGTCACGTCTAGCTACTGCCTCGGAATTCAAGGGGCTACTTTAGGAGCAATTATCTTGTTTACTAAAACTGAATACCTTGCTATCTCTTTGATACATTTTTACAAAGCTGAATTAAAATGGTATAAATTAAATCACTTTGACACAGTCAGACACAGTCCAGCGATCGCGACAGACGCAGTCCCATGGGGACATGGCTGTCATGGTGTGGAAGTGATAGAAATGAAAACATGTATGGATCTGTCACAGGAGCTGGTGAGGCTGATGGGTGTGTGGGTGGCCACTGTTTGCTCTCTGCTTGTCACAGCCTCTTGTTCAGGGCTTGATCAGGGAGGTGTGTGTGTGTGTGTGTGTGGTCACACCCATCTCAGCAGATCTGTCAGCTTTCCCGCTTTTGTTAGAGGGTGATATCATGCTTCCTGGGGGGAGCTCTGGAAGACAATGAGCAGCCACTTTCCTCTAGAATAATCAACCTCTGGATTACAAAATTTGTGAAAGATTGACTGGTATTCTTAACTATGTTGCTCCTTTTACGCTATGTGGATACGCTGCTTTAATGCCTTTGTATCATGCTATTGCTTCCCGTATGGCTTTCATTTTCTCCTCCTTGTATAAATCCTGGTTAGTTCTTGCCACGGCGGAACTCATCGCCGCCTGCCTTGCCCGCTGCTGGACAGGGGCTCGGCTGTTGGGCACTGACAATTCCGTGGGGATCCTGTTTATTTGTGAAATTTGTGATGCTATTGCTTTATTGTAACCATCTAGCTTTATTTGTGAAATTTGTGATGCTATGCTTTATTTGTAACCATTATAAGCTGCAATAAAACAAGTTAACAACAACA |
| **AAV-shENG** | Target sequence | CTGGTCCTCGTTTCGAACAAA |
|  | shRNA oligo sequence | CGAACTGGTCCTCGTTTCGAACAAACTCGAGTTTGTTCGAAACGAGGACCAGG |
|  | AAV sequence | TTTCCACGTTCTCTCGGCATGGACGAGCTGTACAAGTGAGCGAATTGAATGAGGCTTCAGTACTTTACAGAATCGTTGCCTGCACATCTTGGAAACACTTGCTGGGATTACTTCTTCAGGTTAACCCAACAGAAGGCTCGAGAAGGTATATTGCTGTTGACAGTGATTCGAACTGGTCCTCGTTTCGAACAAACTCGAGTTTGTTCGAAACGAGGACCAGGCTAGCTACTGCCTCGGAATTCAAGGGGCTACTTTAGGAGCAATTATCTTGTTTACTAAAACTGAATACCTTGCTATCTCTTTGATACATTTTTACAAAGCTGAATTAAAATGGTATAAATTAAATCACTTTGACACAGTCAGACACAGTCCAGCGATCGCGACAGACGCAGTCCCATGGGGACATGGCTGTCATGGTGTGGAAGTGATAGAAATGAAAACATGTATGGATCTGTCACAGGAGCTGGTGAGGCTGATGGGTGTGTGGGTGGCCACTGTTTGCTCTCTGCTTGTCACAGCCTCTTGTTCAGGGCTTGATCAGGGAGGTGTGTGTGTGTGTGTGTGTGGTCACACCCATCTCAGCAGATCTGTCAGCTTTCCCGCTTTTGTTAGAGGGTGATATCATGCTTCCTGGGGGGAGCTCTGGAAGACAATGAGCAGCCACTTTCCTCTAGAATAATCAACCTCTGGATTACAAAATTTGTGAAAGATTGACTGGTATTCTTAACTATGTTGCTCCTTTTACGCTATGTGGATACGCTGCTTTAATGCCTTTGTATCATGCTATTGCTTCCCGTATGGCTTTCATTTTCTCCTCCTTGTATAAATCCTGGTTAGTTCTTGCCACGGCGGAACTCATCGCCGCCTGCCTTGCCCGCTGCTGGACAGGGGCTCGGCTGTTGGGCACTGACAATTCCGTGGGGATCCTGTTTATTTGTGAAATTTGTGATGCTATTGCTTTATTGTAACCATCTAGCTTTATTTGTGAAATTTGTGATGCTATGCTTTATTTGTAACCATTATAAGCTGCAATAAAACAAGTTAACAACAACA |

**Supplementary Table 4. Reagents used for the study**

| **Name** | **Catalog no.** | **Manufacturer** | **Country** |
| --- | --- | --- | --- |
| Alix | ab275377 | Abcam | USA |
| AQP4 | ab150078 | Abcam | USA |
| CD63 | ab134045 | Abcam | USA |
| GAPDH | ab181602 | Abcam | USA |
| GFAP | ab4648 | Abcam | USA |
| Mouse monoclonal anti-ENG | ab156756 | Abcam | USA |
| Rabbit monoclonal anti-ENG | ab221675 | Abcam | USA |
| Goat Anti-mouse IgG H&L (Alexa Fluor® 555) | ab150118 | Abcam | USA |
| Goat Anti-mouse IgG H&L (Alexa Fluor® 647) | ab150119 | Abcam | USA |
| Goat Anti-rabbit IgG H&L (Alexa Fluor® 555) | ab150078 | Abcam | USA |
| Goat Anti-rabbit IgG H&L (Alexa Fluor® 647) | ab150083 | Abcam | USA |
| HSP70 | ab51052 | Abcam | USA |
| Iba1 | ab178847 | Abcam | USA |
| MCAAD-3 | ab216983 | Abcam | USA |
| TGFBRI | ab235578 | Abcam | USA |
| TGFBRII | ab269279 | Abcam | USA |
| TSG101 | ab125011 | Abcam | USA |
| VIM | ab194719 | Abcam | USA |
| ZO-1 | ab96587 | Abcam | USA |
| ALZET® Micro-Osmotic pumps | MODEL1004 | ALZET | USA |
| Angiotensin II | A1042 | APExBIO | USA |
| Protease inhibitor cocktail | K1007 | APExBIO | USA |
| Phosphatase inhibitor cocktail | K1012 | APExBIO | USA |
| SB 431542 | A8249 | APExBIO | USA |
| APP/β-Amyloid (NAB228) | 2450S | Cell Signaling Technology (CST) | USA |
| GFAP | 3670T | Cell Signaling Technology (CST) | USA |
| Phospho-Smad3 (Ser423/425) | C25A9 | Cell Signaling Technology (CST) | USA |
| Smad3 | C67H9 | Cell Signaling Technology (CST) | USA |
| Amiloride | HY-B0285 | Medchemexpress | USA |
| Carotuximab (TRC105) | HY-P99494 | Medchemexpress | USA |
| GW4869 | HY-19363 | Medchemexpress | USA |
| SB431542 | HY-10431 | Medchemexpress | USA |
| Lipopolysaccharides from E. coli O111:B4 | L2630 | Sigma-Aldrich | USA |
| PKH26 Red Fluorescent Cell Linker Kit | PKH26GL | Sigma-Aldrich | USA |
| BCA protein quantitative kit | Prod 23227 | Thermo fisher scientific | USA |
| DMEM | 11965092 | Thermo fisher scientific | USA |
| 0.25% EDTA Trypsin | 25200072 | Thermo fisher scientific | USA |
| Human endoglin ELISA kit | EHENG | Thermo fisher scientific | USA |
| FBS qualified Australia origin | 10099141C | Thermo fisher scientific | USA |
| Glutamax, 100X | 35050061 | Thermo fisher scientific | USA |
| HBSS | 14175095 | Thermo fisher scientific | USA |
| Lipofectamine™ 3000 | L3000015 | Thermo fisher scientific | USA |
| Penicillin-streptomycin mix | 15140122 | Thermo fisher scientific | USA |
| PBS Ca^2+^ Mg^2+^ Free, pH 7.4 | 70011051 | Thermo fisher scientific | USA |
| Pierce™ Classic Magnetic Bead IP/Co-IP Kit | 88804 | Thermo fisher scientific | USA |
| Pierce™ IP lysis buffer | 87787 | Thermo fisher scientific | USA |
| PrimeScript RT Master Mix | RR036A | TaKaRa | Japan |
| TB Green Premix Ex Taq | RR420A | TaKaRa | Japan |
| BeyoECL Moon | P0018FS | Beyotime | China |
| Enhanced Cell Counting Kit-8 | C0042 | Beyotime | China |
| DEPC water (DNase, RNase free) | R0022 | Beyotime | China |
| DAPI | C1006 | Beyotime | China |
| HRP-labeled Goat Anti-Rabbit IgG (H+L) | A0208 | Beyotime | China |
| Single up™ Immunofluorescence antibody dilute solution | P0277 | Beyotime | China |
| RIPA buffer | P0013B | Beyotime | China |
| Quick block™ Western antibody dilute solution | P0256 | Beyotime | China |
| Total RNA Extraction Kit | BSC52M1 | BioFlux | China |
| HCMEC/D3 | GNHu68 | Shanghai Institute of Biological Sciences | China |
| Human amyloid beta peptide 1-42, Aβ1-42 ELISA Kit | CSB-E10684h | CUSABIO | China |
| Human amyloid beta peptide 1-40, Aβ1-40 ELISA Kit | CSB-E08299h | CUSABIO | China |
| bEnd.3 | FH0356 | FuHeng Biology | China |
| EVs isolation kit | MA0402 | Meilunbio | China |
| Mouse VEGF ELISA Kit | 70-EK283/2 | MultiSciences | China |
| Mouse IL-3 ELISA Kit | 70-EK203 | MultiSciences | China |
| Mouse IL-6 ELISA Kit | 70-EK206/3 | MultiSciences | China |
| Human ENG-overexpression plasmid | HG10149-CH | SinoBiological | China |
| Recombinant Human IL-6 protein | GMP-10395-HNAE | SinoBiological | China |
| Recombinant Human Endoglin protein | 10149-H02H | SinoBiological | China |
| Recombinant Human TNF-α protein | 10602-HNAE | SinoBiological | China |
| Mouse endoglin ELISA kit | SEA980Mu | USCNK | China |
| Mouse S100B ELISA kit | SEA567Mu | USCNK | China |
| DyLight 594 Labeled Tomato Lectin | MP6313 | Jinpan Biotech | China |
| TRITC-Dextran dye | R-FD-039 | Xarxbio | China |

**Supplementary Table 5. Primer sets used for constructs**

| **No.** | **Gene Name** | **Primer set** | **Sequences (5'-3')** | **Manufacturer** |
| --- | --- | --- | --- | --- |
| 1 | mENG | Mouse Endoglin F' | CAGGCTGAAGACACTGACGAC | Sangon Biotech |
|  |  | Mouse Endoglin R' | GAAGTGCGGGCTGAGGTAG |  |
|  |  |  |  |  |
| 2 | hENG | Human Endoglin F' | CGAGGTGACATATACCACTAGCC | Sangon Biotech |
|  |  | Human Endoglin R' | GTTTACACTGAGGACCAGAAGCA |  |
|  |  |  |  |  |
| 3 | Ctsb | Mouse Cathepsin B F' | AAGCCTTCCTTCCACCCG | Sangon Biotech |
|  |  | Mouse Cathepsin B R' | GTCCTCACCGAACGCAAC |  |
|  |  |  |  |  |
| 4 | mGAPDH | Mouse Glyceraldehyde-3-Phosphate Dehydrogenase F' | GAGTGTTTCCTCGTCCCGTAG | Sangon Biotech |
|  |  | mouse Glyceraldehyde-3-Phosphate Dehydrogenase R' | AGGTCAATGAAGGGGTCGTT |  |
|  |  |  |  |  |
| 5 | hGAPDH | Human Glyceraldehyde-3-Phosphate Dehydrogenase F' | GTCAGCCGCATCTTCTTTTG | Sangon Biotech |
|  |  | Human Glyceraldehyde-3-Phosphate Dehydrogenase R' | GCGCCCAATACGACCAAATC |  |
|  |  |  |  |  |
| 6 | GFAP | Mouse Glial fibrillary acidic protein F' | GCGGGATGGAGAGGTCATTA | Sangon Biotech |
|  |  | Mouse Glial fibrillary acidic protein R' | GCGGAGCAACTATCCTGCTT |  |
|  |  |  |  |  |
| 7 | IL-3 | Mouse Interleukin-3 F' | ACTGTTGCCTGCCTACATCTGC | Sangon Biotech |
|  |  | Mouse Interleukin-3 R' | TGCGGGCTGAGGTGGTCTA |  |
|  |  |  |  |  |
| 8 | IL-6 | Mouse Interleukin-6 F' | GAGGATACCACTCCCAACAGACC | Sangon Biotech |
|  |  | Mouse Interleukin-6 R' | AAGTGCATCATCGTTGTTCATACA |  |
|  |  |  |  |  |
| 9 | Serpina3N | Mouse Serpina3N F' | GGCAACACCCTGGAAGAGATT | Sangon Biotech |
|  |  | Mouse Serpina3N R' | CTGGTCCTTTGGCTGGTTGA |  |
|  |  |  |  |  |
| 10 | VEGF | Mouse Vascular endothelial growth factor F' | AAAGCGCAAGAAATCCCGTC | Sangon Biotech |
|  |  | Mouse Vascular endothelial growth factor R' | AAAGCGCAAGAAATCCCGTC |  |
|  |  |  |  |  |
| 11 | VIM | Mouse Vimentin F' | ACCTTGAACGGAAAGTGGAATC | Sangon Biotech |
|  |  | Mouse Vimentin R' | GGCAGCCACGCTTTCATACT |  |
