# Supplementary material for: Endothelium-specific endoglin triggers astrocyte reactivity via extracellular vesicles in a mouse model of Alzheimer’s disease
Source: Mol Neurodegener. 2025 Jul 23;20:84. doi: 10.1186/s13024-025-00875-4 (PMC12285072; doi:10.1186/s13024-025-00875-4)

UNCROPPED WESTERN BLOTS

Figure 3g

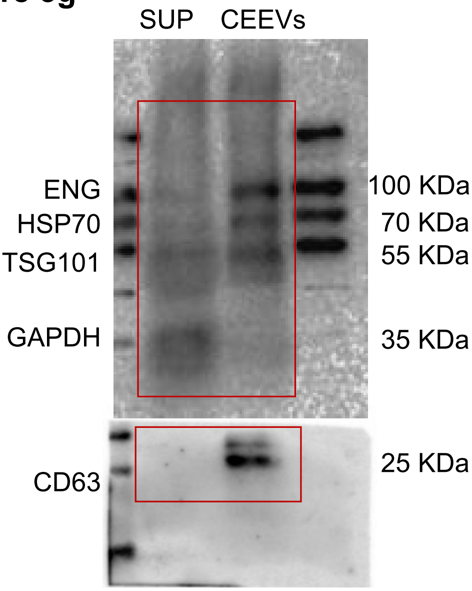

Figure 3h

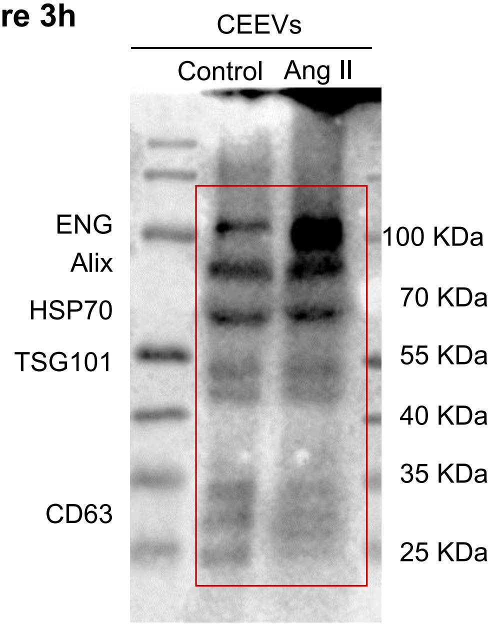

Figure 4a

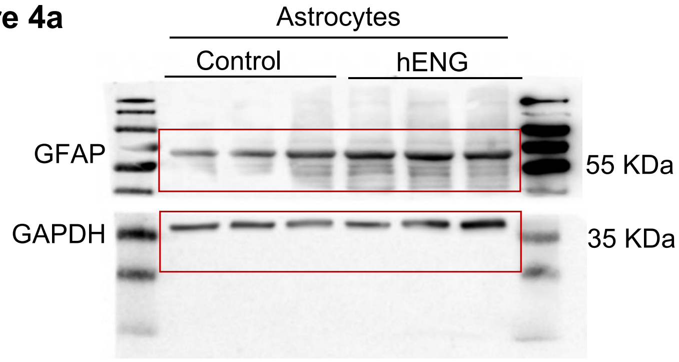

Figure 4b

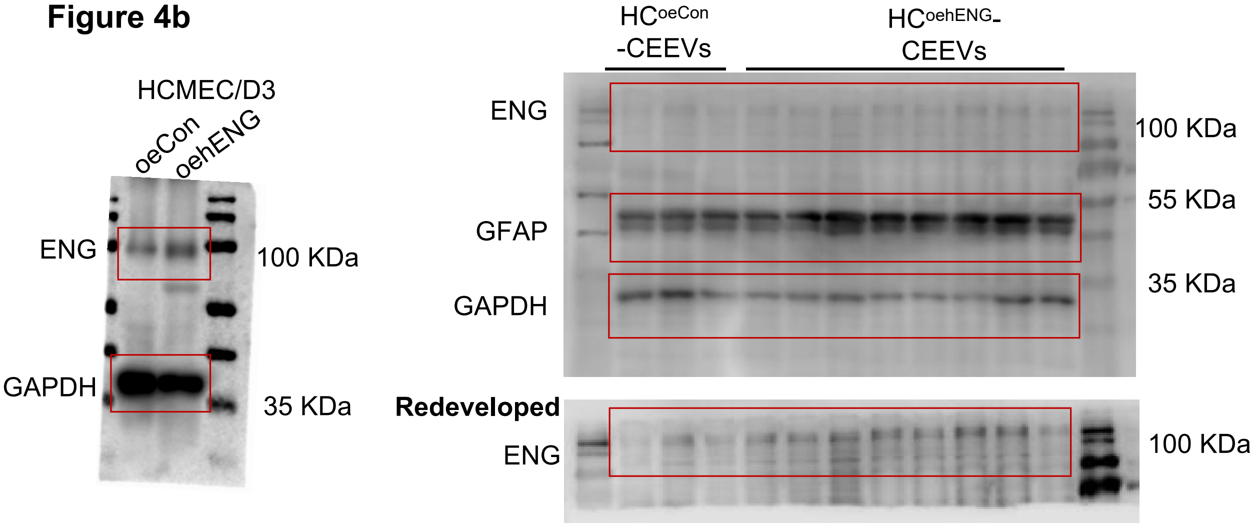

Figure 4d

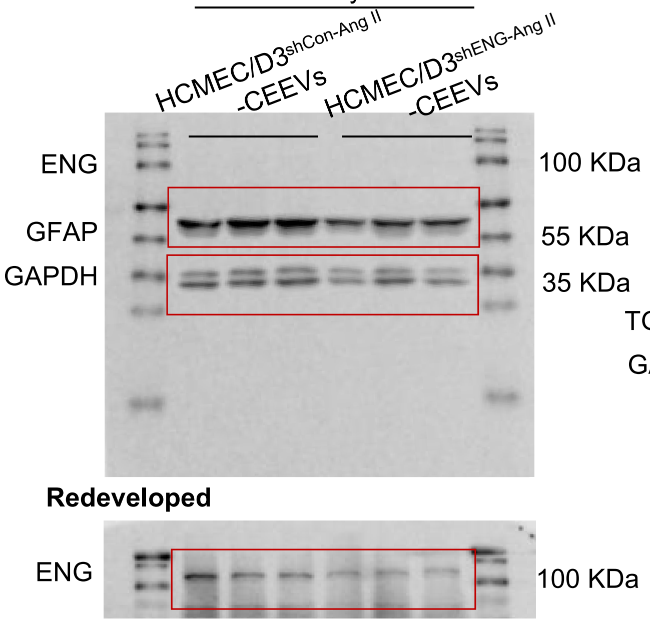

Figure 5a

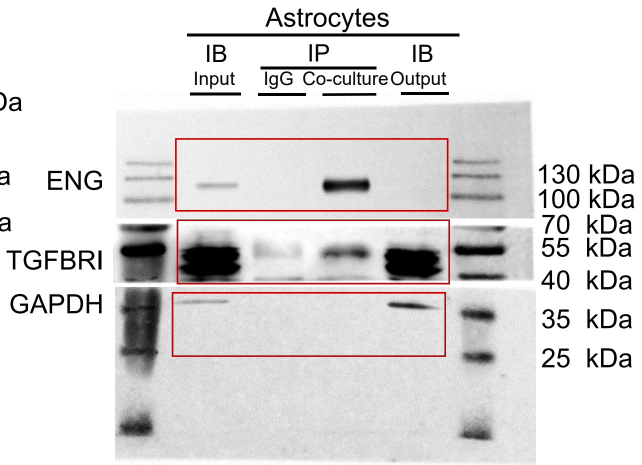

Figure 5c

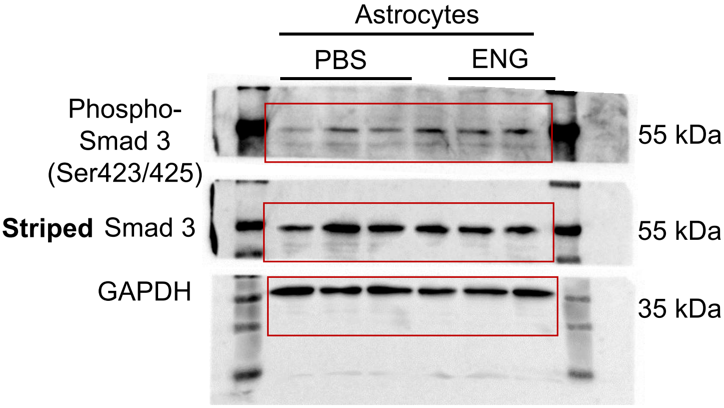

Figure 6b

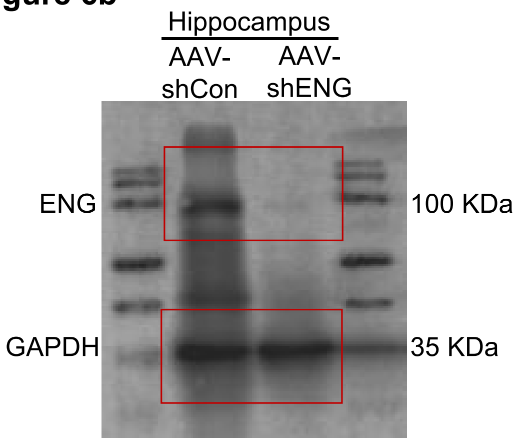

Supplementary Figure 1h

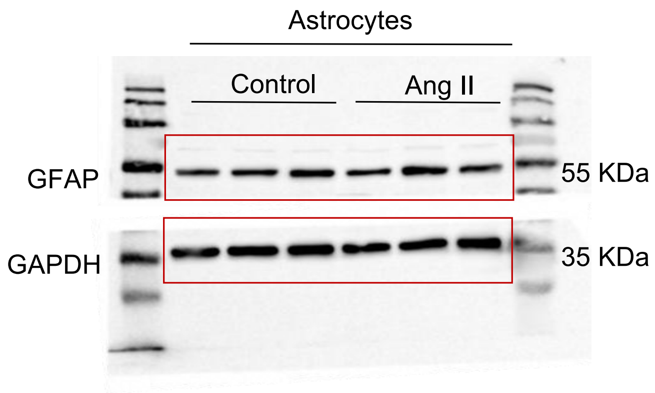

Supplementary Figure 2e

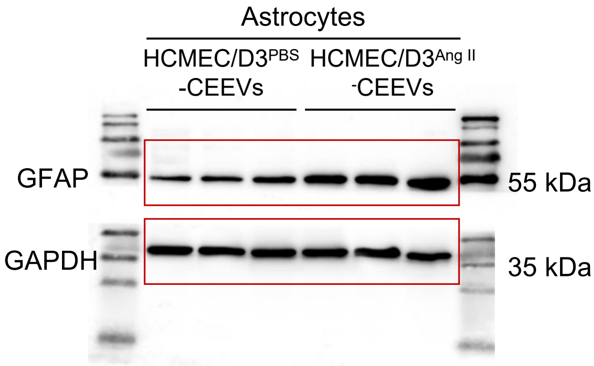

Supplementary Figure 2f

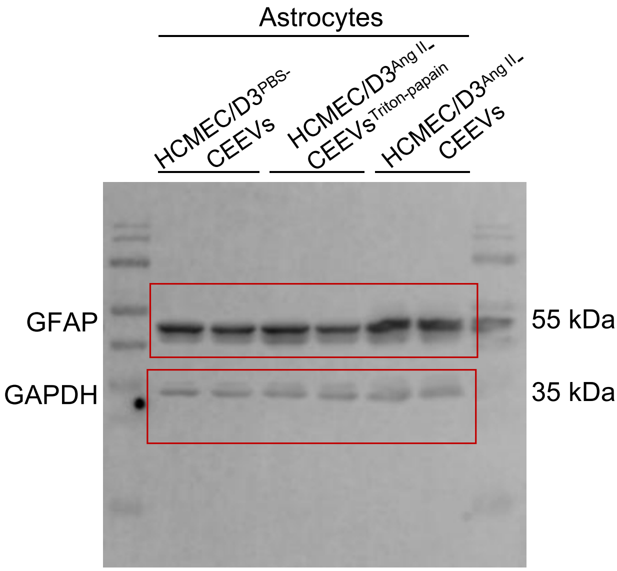

Supplementary Figure 5c

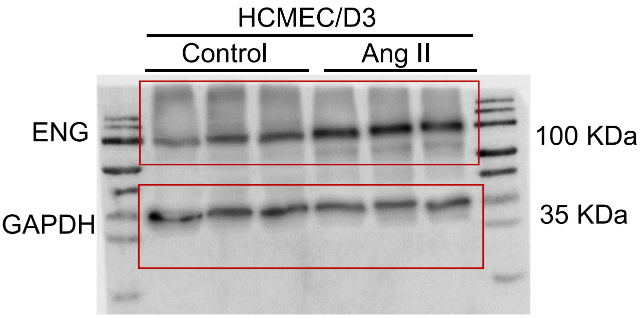

Supplementary Figure 6e

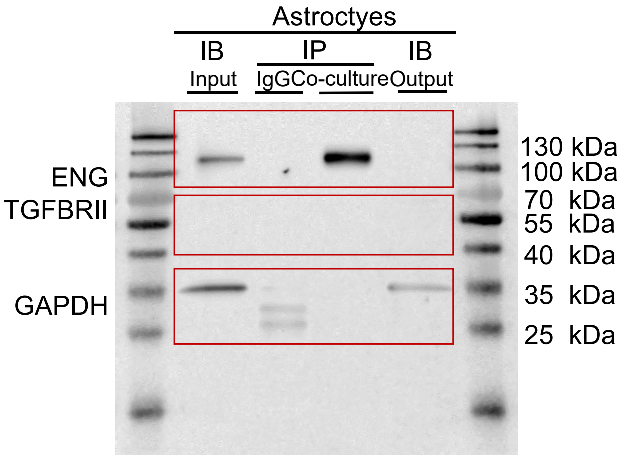

Supplementary Figure 3e

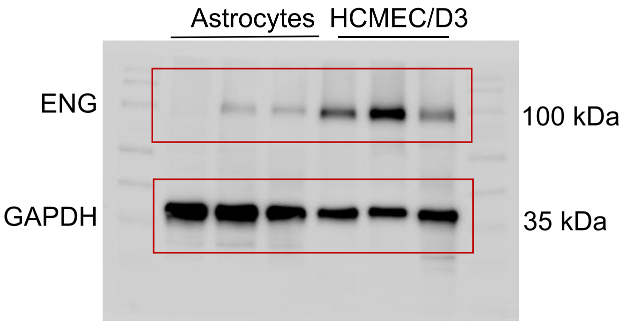

Supplementary Figure 6d

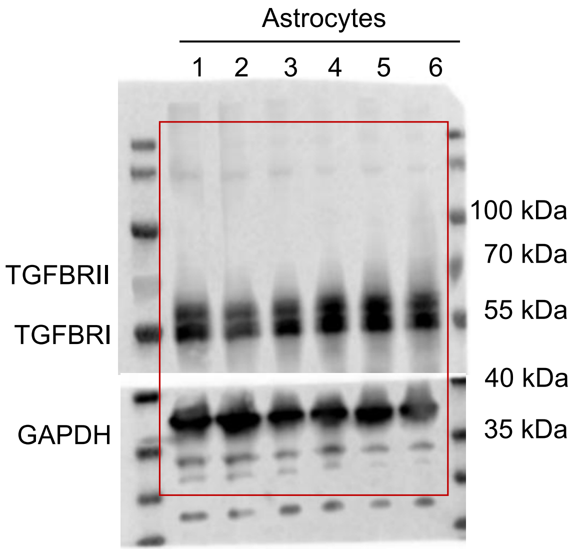

Supplementary Figure 7b

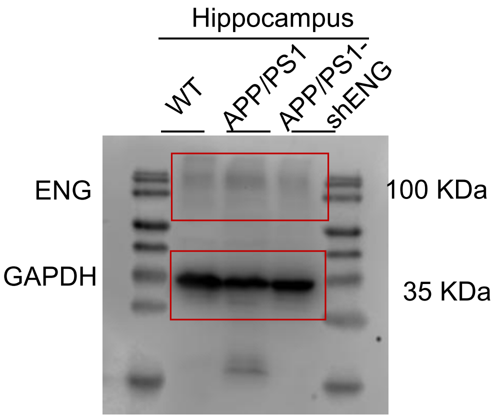

Supplement: Supplementary file 13 — Supplementary Material 13 [file 13024_2025_875_MOESM13_ESM.pdf]
